# Supplementary material for: Capturing the songs of mice with an improved detection and classification method for ultrasonic vocalizations (BootSnap)
Source: PLoS Comput Biol. 2022 May 12;18(5):e1010049. doi: 10.1371/journal.pcbi.1010049 (PMC9098080; doi:10.1371/journal.pcbi.1010049)
Supplement: S1 Text — (DOCX) [file pcbi.1010049.s006.docx]

To our knowledge, only a few studies have used supervised methods for classifying mouse USVs: (1) Vogel et al. [1] classified USVs from C57BL/6J mice into 9 classes, including ‘s’, ‘ui’, ‘c’, ‘f’, ‘up’, ‘d’, ‘c2’, ‘c3’, and ‘c’, using Random Forest [2], an ensemble learning classifier of decision trees. To provide input, 104 features had first been extracted for 25-high SNR instances from each class, and their classifier yielded a classification accuracy of 85%. (2) Coffey et al. [3] developed a classifier (in DSQ) based on Convolutional Neural Networks (CNNs) [4], which was trained on 56000 USVs acquired from B6D2F1 mice (MouseTube dataset). The labels produced by an unsupervised k-means clustering algorithm. These clusters were then simply assigned names post-hoc by visually inspecting the corresponding spectrograms. Using interpolated spectrogram images, it categorizes USVs into 5 default classes: ‘split’, ‘ui’, ‘rise’, ‘c’, and ‘c2’. (3) We [5] classified the elements detected from adult wild-derived house mice (*Mus musculus musculus)* into the classes ‘c2’, ‘c3’, USVs without jumps (‘no-jump’), and FP. In this work, the supervised CNNs was trained using 1200 samples and fed by 2D Gammatone filtered spectrograms (GSs), adapted to the frequency range of mice. The evaluation of its performance showed a macro-F1 score of 90±2.7%. (4) Recently, Premoli et al. [6] classified USVs of mice into 10 classes using different machine learning methods. The classes included ‘c’, ‘h’ (i.e., 'c' with additional calls of different frequencies), ‘c2’, ‘up’, ‘d’, ‘ui’, ‘s’, ‘f’, ‘c3’, and ‘composite’ (i.e., two harmonically independent components). They used 48,669 USVs of NF-kB p50 knock-out mice (B6; 129P2-Nfkb 1tm 1 Bal/J) and control wild-type mice (B6; 129PF2). Avisoft was used for USV detection. They compared the performance of CNNs fed by spectrogram images and different classical machine learning algorithms (including support vector machines) fed by 20 features (obtained by Avisoft). They concluded that there is a 'significant' advantage using images, which contain the entire time-frequency information of the spectrogram (78.8% accuracy), rather than a subset of numerical features for classifying USVs (73.9% accuracy).

## References

1. Vogel AP, Tsanas A, Scattoni ML. Quantifying ultrasonic mouse vocalizations using acoustic analysis in a supervised statistical machine learning framework. Scientific reports. 2019;9(1):8100. doi: <https://doi.org/10.1038/s41598-019-44221-3>.

2. Breiman L. Random forests. Machine learning. 2001;45(1):5-32.

3. Coffey KR, Marx RG, Neumaier JF. DeepSqueak: a deep learning-based system for detection and analysis of ultrasonic vocalizations. Neuropsychopharmacology. 2019;44(5):859-68. doi: <https://doi.org/10.1038/s41386-018-0303-6>.

4. Krizhevsky A, Sutskever I, Hinton GE. Imagenet classification with deep convolutional neural networks. Advances in neural information processing systems; 2012.

5. Abbasi R, Balazs P, Noll A, Nicolakis D, Marconi MA, Zala SM, et al. Applying convolutional neural networks to the analysis of mouse ultrasonic vocalizations2019: Universitätsbibliothek der RWTH Aachen. doi: <https://doi.org/10.18154/RWTH-CONV-239263>.

6. Premoli M, Baggi D, Bianchetti M, Gnutti A, Bondaschi M, Mastinu A, et al. Automatic classification of mice vocalizations using Machine Learning techniques and Convolutional Neural Networks. Plos one. 2021;16(1):e0244636. doi: <https://doi.org/10.1371/journal.pone.0244636>.
