# Supplementary material for: Capturing the songs of mice with an improved detection and classification method for ultrasonic vocalizations (BootSnap)
Source: PLoS Comput Biol. 2022 May 12;18(5):e1010049. doi: 10.1371/journal.pcbi.1010049 (PMC9098080; doi:10.1371/journal.pcbi.1010049)
Supplement: S2 Text — (DOCX) [file pcbi.1010049.s007.docx]

**Data and Method**

**Data**

**Subjects**

The subjects were adult wild-derived house mice (*Mus musculus musculus*), F1, F2 or F3 descendants of wild-caught mice trapped at the Konrad Lorenz Institute of Ethology, Vienna, Austria (48°12′38′′ N, 16°16′54′′E). We used wild-derived rather than wild-caught mice to control for age and rearing conditions. Mice were weaned at 21d and kept in mixed-sex groups with ≤4 siblings per cage until the age of 5 weeks (35d). Henceforth, adult males were housed individually to prevent fighting, and females were housed in sister-pairs whenever possible. The mice were housed in standard cages with bedding, nesting material, a nest box, and a cardboard roll. Food and water were provided *ad libitum*. Housing facilities were kept in standard conditions (22 ± 2 °C and a 12:12 h white light: red light cycle, lights off at 15:00). All recordings were conducted after 15:00 when the mice are most active. We also used recordings of laboratory mice (strain B6D2F1/J) from MouseTube [1].

**Datasets**

Our analyses were conducted using 169 sound files of 48 mice from four different datasets which were recorded in three different contexts or retrieved from MouseTube, respectively. These recordings were either used for development (DEV) or evaluation (EV) of the new method.

The development (DEV) was conducted using sound files of 44 individual wild-derived mice from two different datasets and experiments. The first dataset in the present study consisted of 14 recordings of 10 min duration (each) from F1 mice (subjects: n= 11 males and 3 females; mean ± SD age: 204 ± 17 d; stimulus females: n = 11 and age: 181 ± 15 d), which had been socially primed by a short direct interaction with a female 1d before the recordings (n = 10 priming females, mean ± SD age: 184 ± 16 d) [2]; sex differences reported in [2]; results of priming effects reported in Zala et al. [3]). The second dataset consisted of 10 min recordings of 30 wild-derived (F2) male mice (mean± SD age: 220± 25 d; n = 30 males; and 217 ± 30 d, n = 60 females) recorded twice over two consecutive days. The dataset included 150 sound files from 30 mice recorded over 2 days: 100 sound files of 1 min duration (10 sound files x 5 mice x 2 days = 100 files), due to setting adjustments, and 50 files of 10 min duration (1 sound files x 25 mice x 2 days = 50 files).

The evaluation (EV) was conducted using 5 arbitrarily selected files from the third and fourth datasets. The third dataset consisted of a subset (n=3 soundfiles of 5 min duration) from recordings of wild-derived mice during stimulation with a female odor stimulus [4]. USVs were recorded from adult males (F3, generation, n = 2 males; mean ± SD age: 355 ± 65 d) recorded three times over three consecutive weeks (see below). The fourth dataset consisted of 2 arbitrarily selected sound files of 5 min duration from 168 recordings of laboratory mice (B6D2F1 mice), which were retrieved from MouseTube [1].

**Recording procedures and apparatus (socio-sexual contexts)**

The mice for the first dataset were recorded for 10 min while presented with an unfamiliar stimulus female on the opposite side of a partition, which allowed visual and olfactory stimuli but not direct contact (see details in [5]). A condenser ultrasound microphone (Avisoft Bioacoustics/CM16/CMPA) was positioned 10 cm above the subject's compartment and was connected to an UltraSoundGate 116-200, Avisoft Bioacoustics, Germany.

The mice for the second dataset were recorded for 10 min while separated from a female stimulus by a perforated partition, and then the divider was removed allowing males to interact with the stimulus female and they were recorded for 10 min (as described in [6]). The two mice were then separated again by the divider and recorded for an additional 5 min. An ultrasound microphone (USG Electret Ultrasound Microphone, Avisoft Bioacoustics / Knowles FG) was positioned 10 cm above the male’s compartment and connected to an A/D-converter (UltraSoundGate 416Hb, Avisoft Bioacoustics). This entire procedure was repeated and conducted on the next day with another unfamiliar stimulus female. This procedure allowed us to monitor changes in vocalizations as courtship progressed over time, and the mice also obtained socio-sexual contact and experience through indirect and direct interactions. We recently found that mice significantly increased the amount of USVs (vocal performance) and the number of syllable types (vocal repertoire) after sexual priming [3] and after the partition was removed and they began interacting directly [6]. For the second dataset of this study, we only used recordings during the first 10 min (with the divider) on both days (before and after sexual experience). All recordings for both datasets were conducted inside a recording chamber lined with acoustic foam.

The mice for the third dataset were recorded in a cage with bedding without any stimulus for 5 min (pre-stimulation phase), and then again for an additional 10 min while presented with female urine stimulus (as described in [4]). The urine was a 60 µl pool of thawed female urine (from 3 different unfamiliar females) presented on a cotton swab attached to the cage lid. Mice were recorded in a separate room with no observers or other animals present. This procedure was repeated for each male over 3 consecutive weeks, resulting in a total of 66 recordings. For USV recordings, an ultrasound microphone (USG Electret Ultrasound Microphone, Avisoft Bioacoustics / Knowles FG) was placed 10 cm above the cage and connected to an A/D converter (UltraSoundGate 416Hb, Avisoft Bioacoustics). For each male, the recording of the 10 min stimulus presentation was saved as two separate 5 min sound files to facilitate the processing of single sound files. The 3 arbitrarily selected 5 min sound files used for the third dataset in this study were all recorded during the urine stimulation.

The fourth dataset retrieved from MouseTube [1] originally included 10 min recordings of 12 adult male mice. Mice had 5 min control recordings during the habituation period without any stimulus inside a clean cage. Then, the males were recorded when exposed to 4 different stimuli for 5 min: fresh urine from either females or males, awake adult female, anesthetized adult female, and anesthetized adult male. Each male was exposed to the same stimulus on three consecutive days and to a different stimulus over 4 consecutive weeks (as described in [1]). For the USV recordings, ultrasound microphones (Avisoft Bioacoustics/ CM16/CMPA) were placed over the center of the cage in the recording box and connected to an A/D converter (UltraSoundGate 416H, Avisoft Bioacoustics). Sound files were available on MouseTube and for the fourth dataset of this study 2 sound files were arbitrarily selected from the available soundfiles. All recordings for all datasets were conducted using the RECORDER USGH software (Avisoft-RECORDER Version 4.2) with a sampling rate of 300 kHz and 16-bit format with 256 Hz FFT size for the first 3 datasets, and with a sampling rate of 250 kHz and 16-bit format with 1024 Hz FFT size for the fourth dataset, respectively.

**USV detection and manual classification**

For all datasets, manual USV classification was conducted in STx [7, 8]. Spectrograms in STx were generated using a Hanning window with a range of 50dB, a frame of 4 ms and an overlap of 75% and the spectrogram displayed frequencies up to 150 kHz (Zala et al. [5], [6], and Marconi et al. [4]). USVs and other ambiguous sounds were visually and acoustically inspected. For the first three datasets, USVs were originally labeled according to one of the 12 (first dataset) (adapted from [9], [10], [11], as cited in [5] or 15 (second and third dataset) USV categories (Nicolakis et al. [6], Marconi et al. [4], and Zala et al. [3])) and for the fourth dataset, USVs were labeled according to 6 classes.

For the DEV datasets including the first and second experiment, the USVs were classified (or reduced) into 11 USV categories. The ‘uc’ and some ‘uh’ were excluded from the classification (i.e., 10.5% of the ‘uh’ from the first dataset). However, for the first dataset 89.5% of the ‘uh’ and for the second dataset all ‘uh’ were included in other USV categories if also their spectrographic shape was annotated (e.g., if a USV was originally labeled as ‘uh-up’ because it was over 91 kHz and its shape was ‘up’, it was renamed to ‘up’). The ‘c4’ and ‘c5’ were rarely detected in these sound files and therefore excluded. In summary, the DEV datasets included 11 USV classes (‘up’, ‘d’, ‘c2’, ‘c3’, ‘c’, ‘u’, ‘ui’, ‘f’, ‘s’, ‘us’, and ‘h’) and the FPs (false positives, errors due to the low-SNR recordings) to reach a total of 12 classes. The EV datasets including the third and fourth datasets consisted of 6 classes: ‘c2’, ‘split’ (pool of ‘c3’, ‘c4’, ‘c5’, and ‘h’), ‘c’, ‘ui’, ‘rise’ (pool of ‘up’, ‘d’, ‘f’, ‘s’, ‘us’, and ‘u’), and FP. We created the classes ‘split’ and ‘rise’ because DSQ (DeepSqueak) does not differentiate between individual USVs pooled in these two new classes.

**Gammatone spectrograms preparation**

In speech, unsupervised methods such as Non-negative matrix factorization (NMF) [12, 13] are used to reduce the size of the spectrogram while preserving the time-frequency information. Using NMF, the audio signal spectrogram is approximated using the weighted sum of the basis unit functions, so that the basis unit functions and their weights are non-negative. According to studies, the basis unit functions (or spectral bases) obtained from NMF are very similar to the human cochlea’s biological and perceptual time-frequency resolution [14], as well as perceptual scales, such as the Mel [15] and bark scales [16]. In MUPET, NMF has been applied on the USVs spectrogram to reduce their size along the frequency dimension. The NMF output is the product of spectral bases matrix, which are band-pass filters and are modeled by Gammatone band-pass function, and their weights, which are the spectral magnitude associated with the corresponding filter. To preserve most information and reduce the computational load, the number of spectral bases has been selected as 64. A regression is fitted to the peak frequencies of the base functions to determine the center frequencies and bandwidths of the Gammatone filters, which are as follows:

$$n=\frac{N}{1+e^{-\gamma\left( f_{0}-f \right)}} with \gamma=\frac{2\alpha}{f_{s}} (1)$$

$$B\left( n \right)=\frac{1}{2} \left( f_{n-1}-f_{n} \right) (2)$$

f_s_ is the sampling frequency (i.e., 300 kHz) and N corresponds to the chosen number of filters in the filterbank (i.e., 64). $f_{n-1}$and $f_{n}$ are the central frequency of n-1^th^ and n^th^ Gammatone filter, and B is Gammatone filter bandwidth.

The midpoint frequency (f_0_) and the slope variable (α) were initially obtained from the MUPET script (f_0_=75 kHz and α=14.2). We changed these two parameters (to 68 kHz and 16, respectively), so that all 64 Gammatone filters are generated in the range of 20 kHz to 120 kHz. The variable slope was set based on trial and error as 16. f_0_ is modified based on the mean frequency of the USVs in our data at which most USVs occur. For the calculation of the mean frequency of USVs, we used the frequency track of USVs, which was explained in the Methods section (Input images for the BootSnap). The middle Gammatone filter has the lowest bandwidth (i.e., 0.57 kHz) due to the high number of USVs in midpoint frequency. Other Gammatone filters, which are symmetrically distributed, have higher bandwidth (i.e., between 0.57 kHz and 6.6 kHz) due to the smaller number of USVs in frequencies lower and higher than midpoint frequency.

In the next step, the Gammatone filters are applied as weighted summation kernel to the STFT of USVs subsequently thresholded. This threshold is 10^-3^, so the maximum value between the Gammatone-filtered STFT pixels and the floor noise (10^-3^) was calculated. The output is logarithmically transformed and, then, it is smoothed using an Auto Regression Moving-Average (ARMA) filter **[17]** with order 1.

${\hat{\boldsymbol{C}}}_{\boldsymbol{td}}\mathbf{=}\left\{ \begin{aligned} \frac{\sum_{\boldsymbol{i}\mathbf{=1}}^{\boldsymbol{M}} {\hat{\boldsymbol{C}}}_{\mathbf{(}\boldsymbol{t}\mathbf{-1)}\boldsymbol{d}}\mathbf{+}\sum_{\boldsymbol{j}\mathbf{=0}}^{\boldsymbol{M}} \boldsymbol{C}_{\mathbf{(}\boldsymbol{t}\mathbf{+}\boldsymbol{j}\mathbf{)}\boldsymbol{d}}}{\mathbf{2}\boldsymbol{M}\mathbf{+1}} \boldsymbol{if} \boldsymbol{M}\mathbf{<}\boldsymbol{t}\boldsymbol{\leq}\boldsymbol{T}\mathbf{-}\boldsymbol{M} \\ \boldsymbol{C}_{\boldsymbol{td}} \boldsymbol{otherwise} \end{aligned} \right.$ (3)

The variable $\hat{C}_{td}$ is the spectrum filtered by ARMA, the $C_{td}$ is the spectrum filtered by the Gammatone filterbank, and $M$ is the filter order [18]. Finally, the median filter is applied to remove stationary noise from $C_{td}$. Then zero padding is applied to produce images of USVs with the same size of 401*64. 401 is the width of images, which is related to the maximum duration of USVs (i.e., 200 ms) and 64 is the number of Gammatone filters.

**Classifier**

The following is a brief description of how this model works and how we implemented it.

### Classifier architecture

We used several layers: an input layer, convolution layers, pooling layers, two fully connected (FC) layers, and the output layer. The extraction of information in the CNNs is based on the 2D convolution of kernels and their receptive fields (areas on the input image determined by height and width of the kernel). The 2D convolution is performed by sliding the kernel over the entire image. The resulting matrix is called a feature map ($z_{ij}$):

$$z_{ij}=\sum_{m=0}^{M-1} \sum_{n=0}^{N-1} w_{mn}.x_{\left( i+m+stride-1 \right)\left( j+n+stride-1 \right)}+b_{ij} , (4)$$

$$a_{ij}=\sigma\left( BN\left( z_{ij} \right) \right)$$

Here, $w$ is the convolution kernel matrix, $b$ is the bias, $x$ is the input image, and $M$ and $N$ are the lengths and the width of the kernel. In Equation 4, the stride parameter is specifying the number of pixels to shift the convolution filter. In our approach it is chosen to be 2 for the first layer and 1 for all other convolutional layers. The batch size represents the number of training samples used for training before updating the network weights during one epoch. We trained our network with a batch size of 32 with 200 epochs. The batch-normalization layer (BN) [19] is calculated by normalizing the input of the layer by subtracting the batch mean and dividing it by the batch variance. The nonlinear activation function ($\sigma)$ is applied to each layer output. In the current study, Exponential Linear Unit (ELU) [20] is used for all layers except for the last one (it is softmax for the last one). After applying the activation function on the feature maps, the size of its output is reduced using a pooling layer. We used maximum pooling, which applies no smoothing and retains the key features of the image [21]. Then, the output of the last convolution layer is assigned to the FC layers to allow interactions also on a global level. The activation function of the last layer is the softmax function. The final output is calculated by taking the maximum of the softmax function output. Other activation functions (like ELU) provide an output of real-valued scores that are not conveniently scaled to be used as classifier output. However, the softmax function partitions the probability among the classes helping with the interpretation of the output, without loss of information.

The implementation is based on the Keras library (version 2.2.4) and we run the models training on the Acoustic Research Institute’s clusters with 64 GB RAM, 12-core CPUs, and NVIDIA Titan Xp GPUs, and the other with 64 GB RAM, 8-core CPUs, and NVIDIA GeForce GT GPUs. Data processing and analysis were conducted using Python 3.6, employing NumPy 1.16.2. Also, Sklearn 0.22.1 was used as the framework for model building and training. Figures were produced with Matplotlib 3.1.3.

### Methods for optimization and loss function

In machine learning algorithms, the general aim is to find the optimal weight to minimize the loss function. In this study, we used the categorical cross-entropy (CCE) [22, 23], which computes the dissimilarity between the distribution of the classifier output and the manual labels. For the reduction of the overfitting [24], $L^{2}$ regularization [25], also known as Tychonov or Ridge, is added to CCE as follows,

$$Loss function=CCE+\frac{\lambda}{2m}*{\|w\|}^{2} , where CCE=-\sum_{i=1}^{C} y_{i}(p_{i}) (5)$$

Here, $w$ is the weights matrix of the CNN, $\| .\|$ is the $L^{2}$ norm, the regularization parameter λ is set to 10^-4^ and $m$ is the batch size. The ground truth is denoted by y_i_ while c_i_ denotes the predicted probability of a training sample (i.e., the output of the last layer). c is the number of classes. To optimize the loss function, we used the stochastic gradient descent with Nesterov momentum [26] and we initialized the weights of the convolution and FC layers using the He-initialization [27].

To reduce overfitting and to promote the generalizability of the model [28], we performed the augmentation of the training dataset using random shifts of width and height by 10%. Other augmentation methods such as zooming and normalizing were excluded from this setup as in pilot tests, they increased the validation error of the classifier.

**Training supervised classifier of DSQ:**

In order to fairly evaluate the performance of the DSQ classifier, we have evaluated both the out-of-the-box (pretrained) and retrained models. In the out-of-the-box model, we have used classifier weights obtained from the original DSQ paper. In the retrained model, we used the classifier weights obtained from training the DSQ classifier using DEV data. Both retrained DSQ and BootSnap were trained based on the same 12 target classes. The parameters of DSQ classifier network (including learning rate, number of layers, and the number of epochs) were used unchanged from DSQ 1.0.

### Statistical tests

1. To determine whether two detection tools estimated the duration of USVs with statistically significant different slopes and different intercepts, a permutation test was used. By permuting estimated USV duration for 10000 times, regression line (i.e., y = b0 + b1*x) was fitted between the estimated (x) and observed USV duration (y). The regression line was obtained based on ordinary least squares, which is a maximum likelihood estimator. Then considering the null hypothesis that there is no difference between the slopes (and intercepts), p-values were calculated. These p-values assess whether b0 (and b1) of the two detection methods are significantly different from each other.
2. To estimate the variance of the output of the classifier and IOR, we applied 10000-time bootstrap resampling on the sets of estimates (or classifier output) and ground truth.

### Performance statistics

The performance of the detection tools was evaluated based on TPR and FDR, which are defined as follows:

$$TPR=recall=\frac{tp}{tp+fn}, (6)$$

$$FDR=\frac{fp}{fp+tp} ,$$

where *tp* and *fp* are true and false positives, i.e., the number of correctly and falsely detected samples of USVs, while *tn* and *fn* are true and false negatives, i.e., the correct and false number of omitted USVs.

To evaluate the performance of the classifiers, the macro F1-scores, i.e., the unweighted average of the F1-score of each class was calculated. This metric, unlike accuracy, is not affected by the imbalance distribution of the classes [29]. We also used TPR and FNR (Equation 8) for producing a confusion matrix [30].

$$f1-score=2*\frac{precision*recall}{precision+recall}, where precision=\frac{tp}{tp+fp} (7)$$

$$FNR=\frac{fn}{fn+tp} (8)$$

**References**

1. Chabout J, Sarkar A, Dunson DB, Jarvis ED. Male mice song syntax depends on social contexts and influences female preferences. Frontiers in behavioral neuroscience. 2015;9:76. doi: <https://doi.org/10.3389/fnbeh.2015.00076>.

2. Zala SM, Reitschmidt D, Noll A, Balazs P, Penn DJ. Sex-dependent modulation of ultrasonic vocalizations in house mice (Mus musculus musculus). PloS ONE. 2017;12(12):e0188647. doi: <https://doi.org/10.1371/journal.pone.0188647>.

3. Zala SM, Nicolakis D, Marconi MA, Noll A, Ruf T, Balazs P, et al. Primed to vocalize: Wild-derived male house mice increase vocalization rate and diversity after a previous encounter with a female. PLoS ONE. 2020;15(12):e0242959. doi: <https://doi.org/10.1371/journal.pone.0242959>.

4. Marconi MA, Nicolakis D, Abbasi R, Penn DJ, Zala SM. Ultrasonic courtship vocalizations of male house mice contain distinct individual signatures. Animal Behaviour. 2020. doi: <https://doi.org/10.1016/j.anbehav.2020.09.006>.

5. Zala SM, Reitschmidt D, Noll A, Balazs P, Penn DJ. Automatic mouse ultrasound detector (A-MUD): A new tool for processing rodent vocalizations. PloS ONE. 2017;12(7):e0181200. doi: <https://doi.org/10.1371/journal.pone.0181200>.

6. Nicolakis D, Marconi MA, Zala SM, Penn DJ. Ultrasonic vocalizations in house mice depend upon genetic relatedness of mating partners and correlate with subsequent reproductive success. Frontiers in zoology. 2020;17:1-19.

7. Balazs P, Noll A, Deutsch WA, Laback B. Concept of the integrated signal analysis software system STx. Jahrestagung der Österreichischen Physikalischen Gesellschaft. 2000.

8. Kasess CH, Noll A, Majdak P, Waubke H. Effect of train type on annoyance and acoustic features of the rolling noise. The Journal of the Acoustical Society of America. 2013;134(2):1071-81. doi: <https://doi.org/10.1121/1.4812771>.

9. Musolf K, Meindl S, Larsen AL, Kalcounis-Rueppell MC, Penn DJ. Ultrasonic vocalizations of male mice differ among species and females show assortative preferences for male calls. PLoS one. 2015;10(8). doi: <https://doi.org/10.1371/journal.pone.0134123>.

10. Hoffmann F, Musolf K, Penn DJ. Ultrasonic courtship vocalizations in wild house mice: spectrographic analyses. Journal of ethology. 2012;30(1):173-80. doi: <https://doi.org/10.1007/s10164-011-0312-y>.

11. Hanson JL, Hurley LM. Female presence and estrous state influence mouse ultrasonic courtship vocalizations. PloS one. 2012;7(7):e40782. doi: <https://doi.org/10.1371/journal.pone.0040782>.

12. Févotte C, Idier J. Algorithms for nonnegative matrix factorization with the β-divergence. Neural computation. 2011;23(9):2421-56. doi: <https://doi.org/10.1162/NECO_a_00168>.

13. Lee DD, Seung HS. Algorithms for non-negative matrix factorization. Advances in neural information processing systems; 2001.

14. Fletcher H. Auditory patterns. Reviews of modern physics. 1940;12(1):47. doi: <https://doi.org/10.1103/RevModPhys.12.47>.

15. Stevens SS, Volkmann J, Newman EB. A scale for the measurement of the psychological magnitude pitch. The journal of the acoustical society of america. 1937;8(3):185-90. doi: <https://doi.org/10.1121/1.1915893>.

16. Zwicker E. Subdivision of the audible frequency range into critical bands (Frequenzgruppen). The Journal of the Acoustical Society of America. 1961;33(2):248-.

17. Box G, Jenkins G. Time Series Analysis: Forecasting and Control. Halden-Day, San Francisco. 1970.

18. Van Segbroeck M, Knoll AT, Levitt P, Narayanan S. MUPET—Mouse Ultrasonic Profile ExTraction: A Signal Processing Tool for Rapid and Unsupervised Analysis of Ultrasonic Vocalizations. Neuron. 2017;94(3):465-85. e5.

19. Ioffe S, Szegedy C. Batch Normalization: Accelerating Deep Network Training by Reducing Internal Covariate Shift. International Conference on Machine Learning; 2015.

20. Clevert D-A, Unterthiner T, Hochreiter S. Fast and accurate deep network learning by exponential linear units (elus). arXiv preprint arXiv:07289. 2015.

21. Scherer D, Müller A, Behnke S. Evaluation of pooling operations in convolutional architectures for object recognition. International conference on artificial neural networks; 2010: Springer. doi: <https://doi.org/10.1007/978-3-642-15825-4_10>.

22. Murphy KP. Machine learning: a probabilistic perspective: MIT press; 2012.

23. Goodfellow I, Bengio Y, Courville A, Bengio Y. Deep learning: MIT press Cambridge; 2016.

24. Chen Y, Jiang H, Li C, Jia X, Ghamisi P. Deep feature extraction and classification of hyperspectral images based on convolutional neural networks. IEEE Transactions on Geoscience and Remote Sensing. 2016;54(10):6232-51. doi: <https://doi.org/10.1109/TGRS.2016.2584107>.

25. Hoerl AE, Kennard RW. Ridge regression: Biased estimation for nonorthogonal problems. Technometrics. 1970;12(1):55-67. doi: <https://doi.org/10.1080/00401706.1970.10488634>.

26. Nesterov Y. A method for unconstrained convex minimization problem with the rate of convergence O (1/k^ 2). Doklady an ussr; 1983.

27. He K, Zhang X, Ren S, Sun J. Delving deep into rectifiers: Surpassing human-level performance on imagenet classification. Proceedings of the IEEE international conference on computer vision; 2015. doi: <https://doi.org/10.1109/ICCV.2015.123>.

28. Chen C, Bai W, Davies RH, Bhuva AN, Manisty CH, Augusto JB, et al. Improving the generalizability of convolutional neural network-based segmentation on CMR images. Frontiers in cardiovascular medicine. 2020;7:105. doi: <https://doi.org/10.3389/fcvm.2020.00105>.

29. Sun Y, Wong AK, Kamel MS. Classification of imbalanced data: A review. International journal of pattern recognition and artificial intelligence. 2009;23(04):687-719. doi: <https://doi.org/10.1142/S0218001409007326>.

30. Sammut C, Webb GI. Encyclopedia of machine learning: Springer Science & Business Media; 2011. doi: <https://doi.org/10.1007/978-0-387-30164-8>.
