# Supplementary material for: Capturing the songs of mice with an improved detection and classification method for ultrasonic vocalizations (BootSnap)
Source: PLoS Comput Biol. 2022 May 12;18(5):e1010049. doi: 10.1371/journal.pcbi.1010049 (PMC9098080; doi:10.1371/journal.pcbi.1010049)
Supplement: S3 Text — (DOCX) [file pcbi.1010049.s008.docx]

Here, we compared the estimated USV duration by USVSEG (using the optimal parameters) and A-MUD with the observed USV duration (i.e., manually checked and corrected USV duration). In wild mice, USVSEG and A-MUD underestimated the duration of USVs. In contrast, the duration of USVs from laboratory mice was significantly overestimated by both methods. The overestimation of the duration of the USVs by both methods is probably because the USVs from laboratory mice were very loud and, in most cases, had a strong echo, so both methods considered these echoes as the USVs themselves. However, for the observed durations, the USVs were shortened to the end of the clear tone of the USVs.


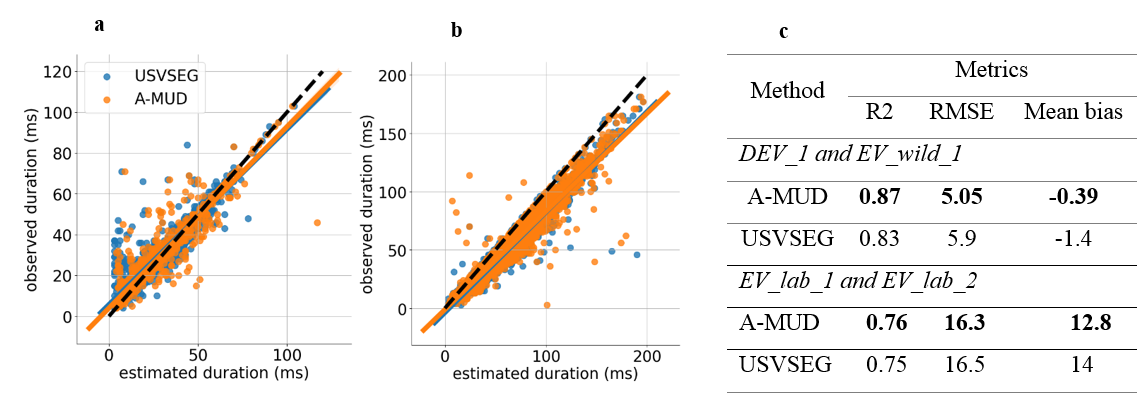


Figure 1. Joint plot between manually corrected (i.e., observed) and estimated duration of detected segments (by A-MUD (orange) and USVSEG (blue)) in (a) DEV_1 and EV_wild_1 data and (b) EV_lab_1 and EV_lab_2 data. (c) Evaluation metrics for the linear regression models between observed and estimated duration of segments. The black dashed line in figures (a) and (b) is the identity line. The evaluation metrics in the table (c) are R-squared (R^2^), root-mean-square error (RMSE), and mean bias between observed and estimated duration of segments. Mean bias is the average difference between the estimated and observed duration of detected segments.

The following table presents the slope and intercept values obtained from the fitted regression line between the estimated (from USVSEG and A-MUD) and observed USV duration.

Table 1. Intercept and slope values obtained from the regression line between the observed and estimated USVs duration for DEV_1 and EV_wild_1 and EV_lab_1 and EV_lab_2 data by A-MUD and USVSEG.

| Parameters | Estimate | Std. error |
| --- | --- | --- |
| *DEV_1 and EV_wild_1* | | |
| Intercept_A-MUD | 3.99 | 0.298 |
| Slope_A-MUD | 0.89 | 0.009 |
| Intercept_USVSEG | 5.7 | 0.30 |
| Slope_USVSEG | 0.86 | 0.009 |
| *EV_lab_1 and EV_lab_2* | | |
| Intercept_A-MUD | -1.1628 | 0.45 |
| Slope_A-MUD | 0.84 | 0.006 |
| Intercept_USVSEG | -3.77 | 0.38 |
| Slope_USVSEG | 0.86 | 0.005 |

Using a permutation test, we found that the difference between slopes (and intercepts) of USV duration estimated by the two tools and real observations are not statistically significant.
