# Supplementary figures and images for: Capturing the songs of mice with an improved detection and classification method for ultrasonic vocalizations (BootSnap)
Source: PLoS Comput Biol. 2022 May 12;18(5):e1010049. doi: 10.1371/journal.pcbi.1010049 (PMC9098080; doi:10.1371/journal.pcbi.1010049)

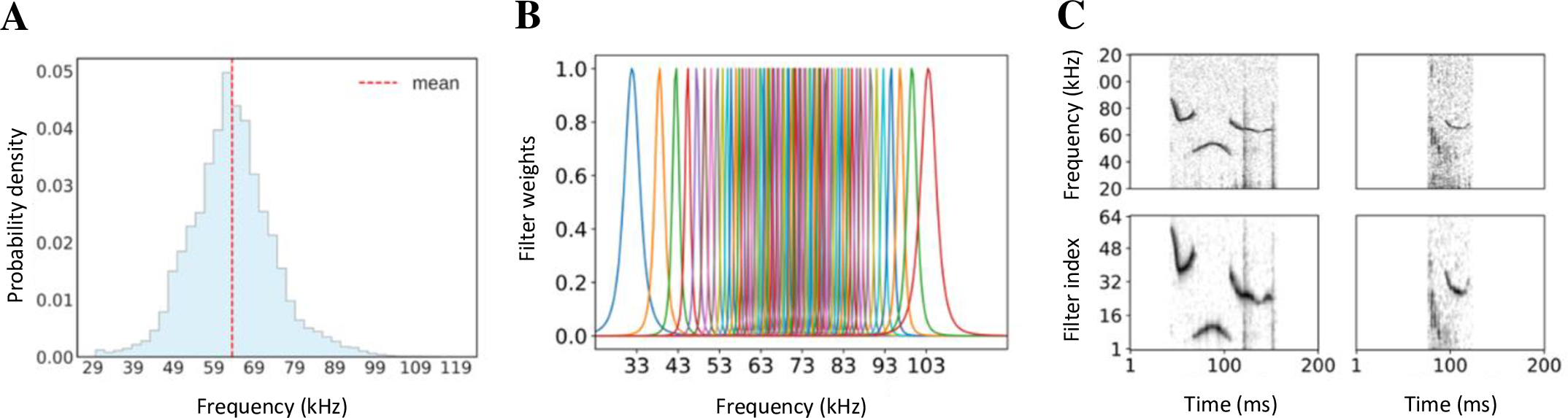

Supplement: S1 Fig — The FT values are related to all detected syllables, omitting false positives. (B) The frequency response of 32 Gammatone filters (we have used 64 filters, but for simplicity 32 filters are plotted here) at the frequency range of 20 kHz to 120 kHz. (C) Two examples of the USVs spectrogram before (top row) and after applying the Gammatone filter and post-processing step (bottom row). This image shows that by applying the preprocessing steps on the spectrogram, the important information of the USVs is not lost, even though the size of the images is reduced from 251 × 401 to 64 × 401. (TIF) [file pcbi.1010049.s009.tif]

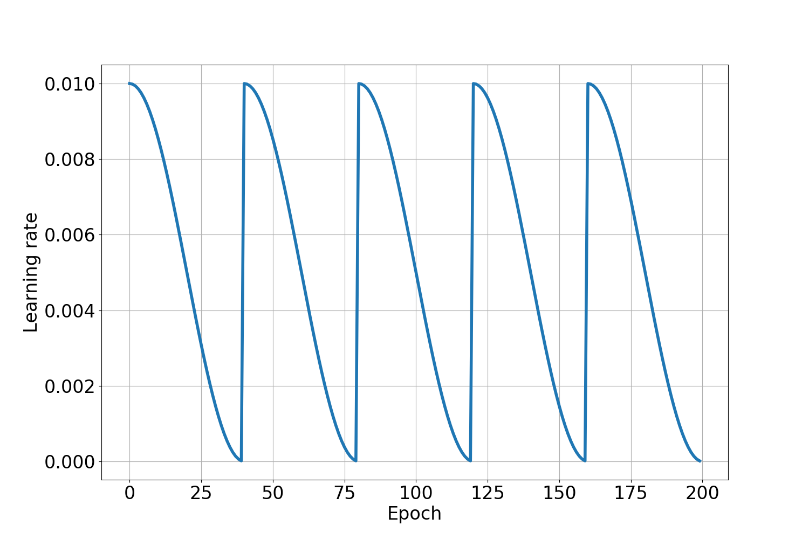

Supplement: S2 Fig — Using this scheme of the learning rate, the final weights of the model at every 40 epochs are the initial weights of the model in the next epoch. In this approach, the CNN weights are saved at the minimum learning rate of each cycle, i.e., at every 40 epochs. (TIF) [file pcbi.1010049.s010.tif]

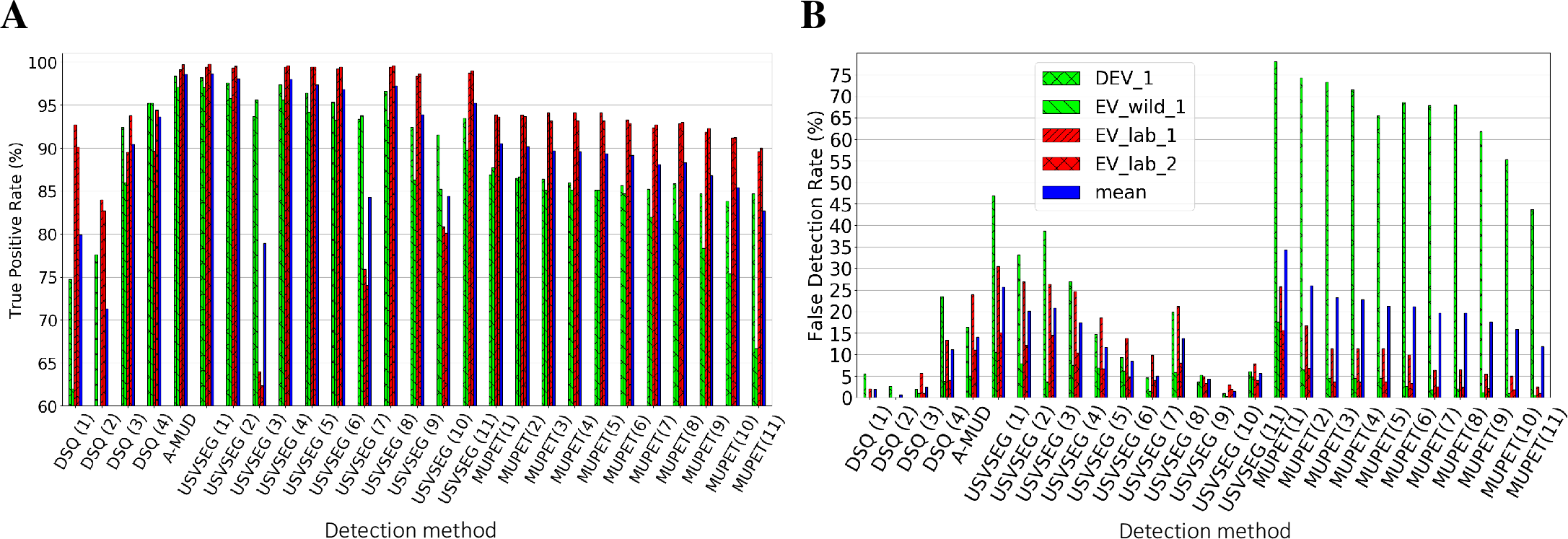

Supplement: S3 Fig — In the main text, we compared the performance of 4 USV detection tools (USVSEG, A-MUD, DSQ, and MUPET) in a setting (i.e., input parameters) of which the selected parameters lead to their best average performance for the four-given data (DEV_1, EV_wild_1, EV_lab_1, and EV_lab_2). Here, we compared the performance of these methods using all the combinations used for their parameters (S2 Table). If we want to compare the best performance of each detection tool with the best performance of others, A-MUD and with a slight difference, USVSEG are in the first and second places, followed by DSQ and MUPET. (TIF) [file pcbi.1010049.s011.tif]

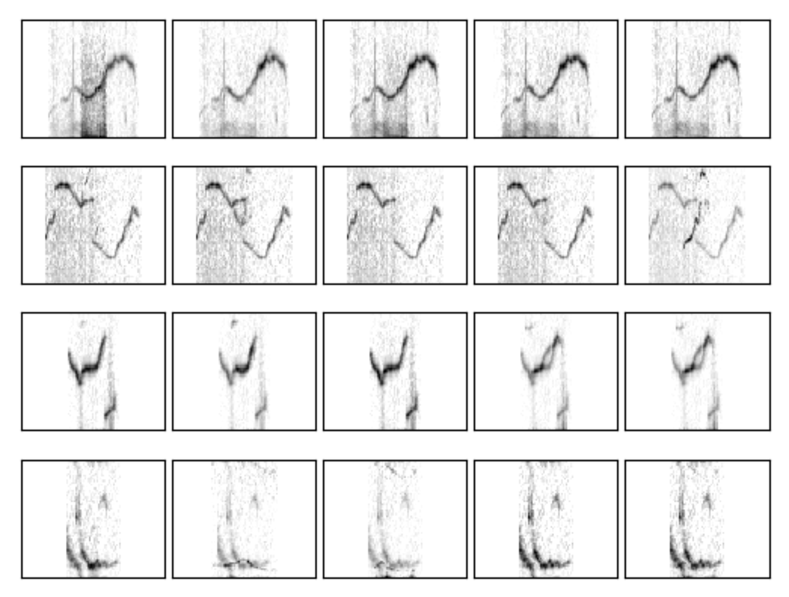

Supplement: S4 Fig — In the model design section, we used various approaches to deal with the problem of the imbalanced datasets, including using original, down-sampled, bootstrapped, and over-sampled data. The following figure presents the over-sampled data by SMOTEENN presented. The first column from the left is the original instance and the next columns are the resampled samples. The first, second, third, and last rows are from the classes ‘c’, ‘c3’, ‘c2’, and ‘h’, respectively. The images produced by the SMOTEENN are very similar to the original data, so, compared to the original data, this method did not help to improve the classifier performance. (TIF) [file pcbi.1010049.s012.tif]

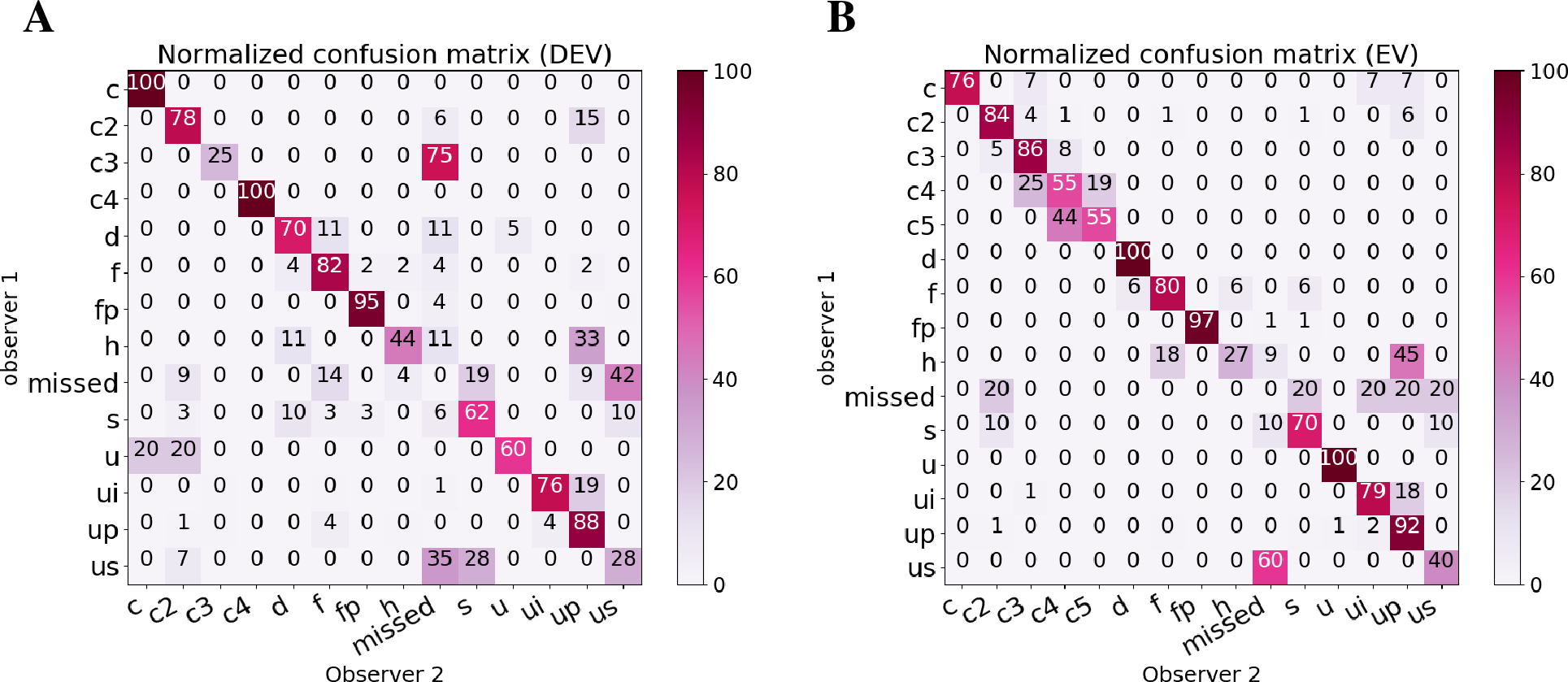

Supplement: S5 Fig — ‘missed’ segments are elements that are manually detected by only one observer. Both figures show high disagreement between the observers for both data in the ‘us’ and ‘h’ classes. In more detail, the amount of reliability in the DEV data in ‘c3’ and ‘u’ classes is very low. Differently, in the EV data, the reliability is less than other classes in ‘c4’ and ‘c5’ classes. (TIF) [file pcbi.1010049.s013.tif]

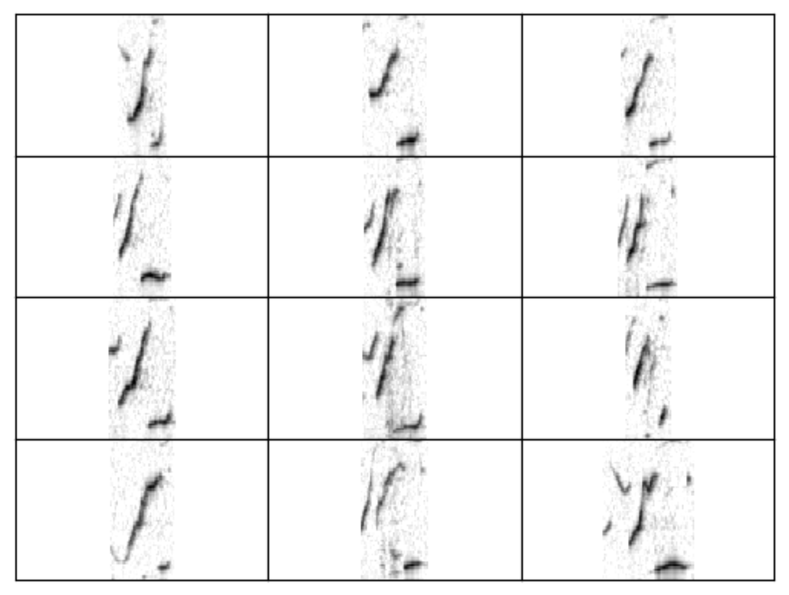

Supplement: S6 Fig — As mentioned in the results section (in the main text), the performance of a model is important when dealing with a new class. Because there was no sample of the ‘c4’ and ‘c5’ classes in the DEV data, we compared the output of the BootSnap and DSQ methods when the two classes were in the EV data. The following figure shows examples of members of these two classes in EV_wild data. BootSnap assigned 68% and 32% of the total members of these two classes to the 2 and 3-jump included USVs, respectively. DSQ assigned the members of the classes ‘c4’ and ‘c5’ mostly to the 2 and 3-jump included USVs and ‘ui’. Although the class ‘ui’ might be relatively similar to the ‘c4’ and ‘c5’ classes based on visual inspection, there is no jump in this class. (TIF) [file pcbi.1010049.s014.tif]
