# Supplementary material for: Capturing the songs of mice with an improved detection and classification method for ultrasonic vocalizations (BootSnap)
Source: PLoS Comput Biol. 2022 May 12;18(5):e1010049. doi: 10.1371/journal.pcbi.1010049 (PMC9098080; doi:10.1371/journal.pcbi.1010049)
Supplement: Attachment — Submitted filename: Response to reviewers.docx [file pcbi.1010049.s023.docx]

**Dear Prof. Stowell and dear Prof. Komarova;**

**We would like to thank you and the reviewers for your thoughtful comments about our manuscript. Here, we address all of the comments, and highlight changes we made in our revised manuscript.**

Editor:

- The reviewers broadly agree on the strengths of the paper but have diverging views on whether the paper presents enough novelty or rigor for publication. The most critical review (R2) raises some significant issues, most of which I concur with.

**As we explain below (R2), our study is the first to comprehensively compare the state-of-the-art USV detection and classification tools, and the first to provide a new model with higher classification generalizability (for new datasets) by using signal processing, machine learning, and statistical methods. And, our main criterion is to minimize the need for user intervention. Here is a summary of the most significant contributions in our manuscript:**

1) **On the generalizability of detection and classification tools:**

**(a) Most USV detection tools are designed (or machines are trained) using data from laboratory mice and a few include wild mice. We evaluated the generalizability of USV detection and classification methods for the first time, and we used both wild and laboratory mouse datasets (lines 154-156 and lines 234 and 235). (b) In previous studies, a few detection tools were examined, however, they were not compared to the “ground truth” (lines 135-141). In our study, we evaluated the performance of the state-of-the-art detection tools using a significant number of observations (lines 157-159). (c) In regard to the performance of the USV classification models, none have compared the performance of their developed model with others (e.g., (Fonseca et al., 2021) and (Premoli et al., 2021) or with ground truth (Coffey et al., 2019) (lines 222-226). We explain how we address this gap (lines 233-236 and sections “Comparing BootSnap and DSQ: transferability to new datasets” and “Comparing BootSnap and DSQ: sensitivity to new classes”).**

2) **Novelty of our classification model:**

**(a) Unlike existing classification models (e.g., (Vogel et al., 2019), (Coffey et al., 2019), and (Premoli et al., 2021)), which only use high-SNR data for training, we used a combination of high- and low-SNR training data. This avoids the degradation of the model performance for the low-SNR test dataset (Wu et al., 2008) (lines 200-204, lines 298-303). (b) For the first time, we developed a classification model with higher generalizability than others, including the state-of-the-art model (DSQ), by combining signal processing and statistical and machine learning algorithms. Gammatone spectrograms are used for reducing the size of inputs, which leads to reduced computational expenses and the possibility of overfitting (lines 321-326). The use of Snapshot ensemble helps to take advantage of ensemble learning without the need to train additional models. We have now provided additional clarifications on lines 212-221 and 419 and 420. Finally, the bootstrap is used to increase (decrease) the randomness (variance) during the model development (lines 392-394). These features all contribute to the better generalizability of our model.**

**(c) To provide a fair comparison between our classification model and DSQ, the state-of-the-art tool, our test comparison is implemented by using an evaluation dataset that is not used in the training phase (lines 233-235, lines 251-259, and lines 635-639).**

**Additional innovations of our study, which we hope will raise the standards for the future, include:**

3) **For the first time, we provide manually labeled evaluation data and our sound files are available on the MouseTube repository.**
4) **For the first time, we report class-wise inter-observer reliability of ground truths (line 434-42638, and lines 873 and 874).**

Reviewers:

**Before addressing the reviewers’ comments, we first summarize the 4 main changes that we made to our revised version of the manuscript:**

1- **We followed the suggestion of R2 and tried different sets of parameters for USVSEG and MUPET, and our changes are in Figure 4 in the main text and Supplementary Figure 3 and Table 1 in the Supplementary File 3 (Figure 4 and 5 and Table 3 in the main text and Figure 3 in the Supplementary of the old manuscript). Figure 4 now shows the best performance of USVSEG and MUPET (in terms of TPR) using new sets of parameter inputs. Please note that our conclusion that A-MUD has better overall performance compared to other tools is still valid.**
2- **We mixed up the definitions of false positive rate (FPR) vs. false detection rate (FDR) in our old manuscript. The number of segments mistakenly detected as USVs to the total number of detected segments was defined as FPR. In the revised manuscript, we now use the correct term for this ratio which is FDR. Apologies for our mistake.**
3- **In Supplementary Figure 3 (of the old manuscript), the best TPR and FDR of MUPET have been erroneously calculated (by using the noise-reduction parameter of 5, minimum syllable duration of 5 ms, and the minimum frequency of 30 kHz) in the following data: DEV_1, EV_lab_1, and EV_lab_2. The TPRs calculated for the above three data-sets changed from 95%, 94%, and 92% to 84%, 91%, and 91% and the FDRs changed from 48%, 3.5%, and 1% to 55%, 5%, and 1.9%. It should be noted that MUPET did not yield the best performance using the setting above, and that we used more inputs for parameters as suggested by R2 to achieve the best results for MUPET. This error did not change the conclusion about the best detection tool. We do apologize for this mistake.**
4- **According to the comment of reviewers 2 and 3, the lines 337 to 397 and lines 467 to 482 of the old manuscript were moved to the Supplementary File 2.**

Reviewer #1:

Abbasi et al. provide an extensive manuscript detailing the comparison of out-of-sample performance of several publicly available algorithms for detection and classification of rodent USVs, tested here in specifically mice recordings. The authors address an important gap in the literature, namely a systematic evaluation of generalization of algorithms that have been trained with restricted datasets (either in SNR, elicitation, age/sex/species). This evaluation can contribute to the most effective uptake of automatic USV detection and classification methods that are robust against variation between recording settings. The authors focus on supervised ML approaches, reporting macro F1 scores for overall comparison and detailed micro F1 scores across different ranges of class-numbers. Importantly, they test their models on gold-standard human annotation, for a completely new dataset, and also provide inter-rater reliability between two human experts on this dataset, as a practical upper limit to classification accuracy. The core of the manuscript is well developed and does not raise major concerns. Methods are described in detail and most metrics use resampling/cross validation to arrive at an estimate of variance.

- The MS could benefit from some style/language editing, and a careful inspection of the in-text references to figures and tables that are sometimes off (e.g., line 1328)

**We thank the reviewer for the comment. We have edited the manuscript, and references to figures and tables were added in the text.**

- I have several suggestions for small improvements: Figure 4, Table 5,6,7 are (mostly) missing variance estimates. Can the authors provide (resampled) variance estimates to support inferential statistical comparisons between groups?

**Thank you for your suggestion. Tables 5, 6, and 7 and Figure 4 were updated by adding the variance applying 10000-time bootstrap resampling on the sets of estimates (or classifier output) and ground truth.**

- Figure 5/table 3 can be improved. it seems the AMUD slope + AMUD intercept SE are off by a misplaced decimal separator in the EV_lab subtables? The significance estimates of the slopes are not particularly interesting. Rather, a permutation test assessing the significance of the difference in slopes (and intercepts) in the USV length estimates might be more informative.

**In the revised manuscript, according to the reviewer’s suggestion, the significance of the difference in slopes (and intercepts) was calculated using permutation. Based on the obtained p-values (*p* > .05), the null hypothesis, i.e., that there is no difference between the slopes (and intercepts) of USVSEG and A-MUD, could not be rejected. This new result is added to the manuscript in the lines 496-498. The explanation of permutation test added in the supplementary file 3.**

- These particular results feel slightly disconnected from the main story and might be delegated to the supplement, unless it can speak to the us vs. s misclassification discussion later on, but that involves an AMUD vs DSQ comparison if I am not mistaken.

**We moved these results to the Supplementary File 3. However, due to the influence of USV duration in the classification, lines 495-501 were added to the manuscript.**

- it is unclear which parameter options have been considered in the hyperparameter optimization of the CNN models. Surely, the authors have used different layer setups, learning rates, dropout percentages. Please report the space of the grid search performed over the hyperparameters, and potentially the DEV validation accuracy attained with some/all combinations.

**We evaluated the model for different inputs of the hyperparameters and the architecture (which were not reported in our manuscript), including the following parameters: the number of convolution layers (3, 4, and 5), the number of filters in each convolution layer (16, 32, 64, and 96), the kernel size in the first convolution layer ((3, 3), (5, 5), (3, 18)), the drop out percentages (0.5, 0.6, 0.7), the size of dense layers (32, 64, 128), and the learning rate (cosine annealing learning rate scheduler** **(Huang et al., 2017), fixed learning rate=10^-3^, and decreasing learning rate=(10^-3^ to 5*10^-6^)). This information is now included on lines 364-369. For brevity, the performance of some combination of the hyperparameters in the model is reported in the Table 4 in the Supplementary.**

Reviewer #2: Synopsis

--------

Many researchers are interested in the automated quantification of mouse vocal behavior (ultrasonic vocalizations or USVs), which is useful for the study of models of various diseases and the neural underpinnings of social behaviors, among other topics. In the typical analysis approach in which single "syllables" or "calls" are segmented and then described quantitatively, there are two chief technical challenges which are both active research areas. First, the researcher must automatically and reliably detect USVs from a stream of audio. Second and more open-endedly, the researcher must produce meaningful and useful quantifications of the resulting USVs. Substantial progress or improved understanding of either subtask would be a valuable contribution to the field.

- The authors present two primary contributions: an empirical comparison of our four previously published USV detection methods and experiments exploring the supervised classification of manually-defined syllable types, which includes a proposed method called “BootSnap.” Unfortunately, I do not believe either contribution provides substantial progress or methodological insight. Therefore, I do not recommend this manuscript for publication.

**We apparently did not clarify enough the novelty and importance of our study in our manuscript. Our study is the first to comprehensively compare the state-of-the-art USV detection and classification tools, and the first to provide a new model with higher classification generalizability (for new datasets) by using signal processing, machine learning, and statistical methods. And, our main criterion is to minimize the need for user intervention. Here is a summary of the most significant contributions in our manuscript:**

5) **On the generalizability of detection and classification tools:**

**(a) Most USV detection tools are designed (or machines are trained) using data from laboratory mice and a few include wild mice. We evaluated the generalizability of USV detection and classification methods for the first time, and we used both wild and laboratory mouse datasets (lines 154-156 and lines 234 and 235). (b) In previous studies, a few detection tools were examined, however, they were not compared to the “ground truth” (lines 135-141). In our study, we evaluated the performance of the state-of-the-art detection tools using a significant number of observations (lines 157-159). (c) In regard to the performance of the USV classification models, none have compared the performance of their developed model with others (e.g., (Fonseca et al., 2021) and (Premoli et al., 2021) or with ground truth (Coffey et al., 2019) (lines 222-226). We explain how we address this gap (lines 233-236 and sections “Comparing BootSnap and DSQ: transferability to new datasets” and “Comparing BootSnap and DSQ: sensitivity to new classes”).**

6) **Novelty of our classification model:**

**(a) Unlike existing classification models (e.g., (Vogel et al., 2019), (Coffey et al., 2019), and (Premoli et al., 2021)), which only use high-SNR data for training, we used a combination of high- and low-SNR training data. This avoids the degradation of the model performance for the low-SNR test dataset (Wu et al., 2008) (lines 200-204, lines 298-303). (b) For the first time, we developed a classification model with higher generalizability than others, including the state-of-the-art model (DSQ), by combining signal processing and statistical and machine learning algorithms. Gammatone spectrograms are used for reducing the size of inputs, which leads to reduced computational expenses and the possibility of overfitting (lines 321-326). The use of Snapshot ensemble helps to take advantage of ensemble learning without the need to train additional models. We have now provided additional clarifications on lines 212-221 and 419 and 420. Finally, the bootstrap is used to increase (decrease) the randomness (variance) during the model development (lines 392-394). These features all contribute to the better generalizability of our model.**

**(c) To provide a fair comparison between our classification model and DSQ, the state-of-the-art tool, our test comparison is implemented by using an evaluation dataset that is not used in the training phase (lines 233-235, lines 251-259, and lines 635-639).**

**Additional innovations of our study, which we hope will raise the standards for the future, include:**

7) **For the first time, we provide manually labeled evaluation data and our sound files are available on the MouseTube repository.**
8) **For the first time, we report class-wise inter-observer reliability of ground truths (line 434-42638, and lines 873 and 874).**

Major Comments

--------------

- In Supplementary Table 2, describing the detection parameters for each detection algorithm, only the four pre-packaged networks are described for DeepSqueak. From what I can tell, the intended use case of DeepSqueak is for the user to train a detection network using their own species- and laboratory-specific data. Simply using the pre-packaged networks without any fine-tuning is not the intended use case of DeepSqueak and will likely present an overly pessimistic picture of its performance as it is used in practice. I consider this a major weakness of the manuscript.

**We are grateful for raising this point, as it helps to better state our main goals, which include providing a classifier that has the highest efficiency with the least user intervention. Many researchers would like to have an "out-of-the-box" detection method. In addition, many final users might not have enough experience to optimize the parameters of a given method. This point has now been added in lines 19 and 20 in the abstract and lines 159-163 in the introduction. The importance of model automation for detection can be seen in the following papers: (Concha‐Miranda et al., 2020; Fonseca et al., 2020; Hernandez-Lallement et al., 2020; Morales-Navas et al., 2020; Odent et al., 2021; Paraouty et al., 2021; Sanchez et al., 2021).** **Please note that only two of these 7 studies re-trained DSQ for USV detection. Most users are unlikely to re-train the model (due to resource constraints) and this point can be seen in the response of Fonseca et al (Mar 31, 2021) to their reviewers (https://elifesciences.org/articles/59161).** **Thus, re-training DSQ contradicts the purpose of our study, and there are several obstacles for re-training DSQ:**

**1) Users need to manually resize the window of each segment and reject or accept detected calls; 2) users need to manually refine the threshold for power or another "score” used for detection; and most importantly 3) changes in the previous two steps will affect the model performance and this requires a significant number of ground truths, and, in most cases, users cannot access to this information.**

**We respectfully disagree with the comment that we provide a pessimistic assessment of DSQ. In our study, we tried to provide a suitable structure for comparing DSQ with other tools. For this purpose, all the evaluation data are taken from data sources that are not involved in the development of any of the studied tools. This point is discussed in lines 153-163.**

- Also, in Supplementary Table 2, there are only two sets of parameters tested for MUPET and three for USVSEG. This is a very small number compared to, say, a standard grid search, which may result in a pessimistic picture of these methods' performance.

**We evaluated the performance of USVSEG and MUPET by 6 sets of their parameters (within the range recommended by the developers of each tool), but in the end, only the two or three best performances were reported in our old manuscript. Following the reviewer’s recommendation, we compared USVSEG and MUPET for 11 sets of parameters and we report the results in our revised manuscript. It is worth noting that we tried to avoid any “pessimistic picture of the methods’ performance”, as for some parameters, the tested inputs were not limited to the recommended intervals, when the inputs, which were out of the range, performed better.** **For example, the TPR of USVSEG reached by A-MUD was 98.6% after setting the parameter “threshold” to 2.5 even though the recommended range is 3.5-5.5. But in terms of FDR, it underperformed A-MUD (25.7% vs. 13.7%). Also, MUPET’s performance improved after setting the noise-reduction parameter to 2. The best TPR increased to 90.5%, but, compared to A-MUD (98.6%), DSQ (93.6%), and USVSEG (98.6%), still had a poorer performance. It is important to note that despite the improvements above, A-MUD still outperformed the other detection tools considering both TPR and FDR. The best performance of the detection tools is shown in Figure 4 and the related explanations are updated (lines 23-25, 477-479, 490-494, 769-773, and 779-786).**

- In the abstract, the authors write "all four methods had comparable rates of detection failure, though A-MUD outperformed the others in terms of true positive rates for recordings with low or high signal-to-noise ratios." This seems to be fairly misleading given that A-MUD clearly has the highest false positive rate among all test sets excluding "DEV_1" (Figure S3).

**We emphasize TPR because the main objective of our study is to remove false positives (FPs) automatically. After setting the parameter “threshold” to 2.5 for USVSEG, the TPR obtained from its best performance equaled A-MUD. However, USVSEG produced a higher mean FDR than A-MUD (25.7% versus 13.7%). One might wonder whether these FPs could be removed automatically in the classification step, however, by examining the output of USVSEG, we found that most of its FPs are fragmented faint USVs, such that they do not resemble FPs and thus must be removed or corrected manually. The abstract is modified as follows:** **A-MUD and USVSEG outperformed the other methods in terms of true positive rates using default and adjusted settings, respectively, and A-MUD outperformed USVSEG when false detection rates were also considered.**

- It is possible that the other models' true positive rates would improve to the level of A-MUD's if their false positive rates were allowed to increase. Trying more sets of parameters for each model, perhaps enough to sketch an ROC curve, would be useful in this comparison. As it stands, I think the evidence for A-MUD's superiority is fairly weak.

**As mentioned earlier, we have examined the performance of the tools for 6 sets of parameter inputs. But for the sake of brevity, we reported only two or three best performances. In the revised manuscript, a larger number of inputs (i.e., 11) was used as parameters, including those increasing the false positive rates. Again, only the best performance is reported in the manuscript and 10 other performances are to be found in the supplementary (please check lines 769-771 and 779-787, supplementary table 3, figure 4, and supplementary figure 3). In general, none of the studied tools could outperform A-MUD after testing more and different parameter inputs.**

- On a related note, I noticed many of the same authors on the references of the development and evaluation datasets and the A-MUD methods paper. Is it possible that the data used to develop the original A-MUD and its successive versions shares characteristics with the datasets used in this paper, for example distinct microphone or cage characteristics or the genetic backgrounds of mice? If so, this would also present a concern in evaluating the methods. If not, a couple sentences clarifying this would be warranted.

**As stated in the results and discussion sections, our focus is evaluating our tool with different data, which include USVs from our lab and others (as stated in lines 142-157 and lines 281-286). All recordings were conducted using microphones which are commonly used to record mouse USVs (Avisoft Bioacoustics) as described in the Supplementary File 2. Furthermore, we used datasets from different contexts (e.g., males with fresh or frozen female urine, males or females with a stimulus female separated by divider) and male or female subjects without or with socio-sexual experience. These contexts and treatments are commonly used when eliciting mouse USVs. We used wild-derived mice of different generations recorded in different contexts and included files from laboratory mice in order to validate our method for a more general use. We now clarified these points in lines 260-268.**

**In total, we used 4 different datasets from which 2 were used for development (DEV) and 2 for evaluation (EV), and for each of them we point out the characteristics, separately:**

**DEV_a: 11 males and 3 females (F1 generation of wild-derived house mice, M. m. musculus) recorded in the presence of a stimulus female through a partition. Subjects had a previous socio-sexual experience (i.e., 5 min direct interactions with a female). Recordings were conducted in recording cages (42.7 x 26.7 x 15 cm) with a condenser ultrasound microphone (Avisoft-Bioacoustics CM16/CMPA) (Zala et al., 2017).**

**DEV_b: 30 males (F2 generation of wild-derived house mice, M. m. musculus) recorded in the presence of a stimulus female through a partition on 2 consecutive days without and then with socio-sexual experience. Recordings were conducted in recording cages (42.7 x 26.7 x 15 cm) with a USG Electret Ultrasound Microphone (Avisoft Bioacoustics / Knowles FG).**

**EV_wild: 3 recordings selected out of a dataset of 22 sexually experienced males (F3 generation of wild-derived house mice, M. m. musculus) recorded in the presence of thawed female urine pool. Recordings were conducted in standard home cages (36.5 x 20 x 14 cm) with a USG Electret Ultrasound Microphone (Avisoft Bioacoustics / Knowles FG) (Marconi et al., 2020).**

**EV_lab: 2 recordings selected out of a dataset of 12 males (laboratory mice strain B6D2F1/J) retrieved from MouseTube. Males were recorded in the presence of fresh female urine in a standard home cage (36.5 x 20 x 14 cm) with a condenser ultrasound microphone (Avisoft-Bioacoustics CM16/CMPA) (Chabout et al., 2015).**

**Furthermore, the laboratory mouse USVs obtained from MouseTube were recorded at a different institution and had nothing in common with the data used in the A-MUD development. So, the good performance of this tool is not biased by the use of similar data. Results show that A-MUD performs far better than other tools. A-MUD was developed based on wild-derived mice, whereas data of laboratory mice were used to develop USVSEG, MUPET, and DSQ (lines 281-284).**

- I am unsure what to take away from the results in Figure 5 regarding the duration of detected syllables. The trendlines of A-MUD and USVSEG look very similar by eye and the R^2 differences are fairly minor and heavily influenced by outliers.

**We thank the reviewer for this correction, as there is no significant difference in how USVSEG and A-MUD estimate the duration of USVs. In further studies (following the suggestion of R1) using permutation, we found that the slopes (and intercepts) between USV duration estimated by the two tools and observations are not statistically different. Therefore, we transferred this figure and related explanations to the Supplementary file 3. Please note that the values in table 1 in supplementary file 3 for USVSEG have been updated due to further testing of parameter sets for USVSEG.**

- I see that the differences are significant if each syllable is treated as independent (Table 3), but the result that A-MUD performs better with wild mice and USVSEG with laboratory mice is anecdotal given only two combined groups of datasets are compared.

**As stated above, we followed the recommendation of R1, and instead of the significance of slope and intercept, in the revised manuscript, we reported the significance of the difference between slopes (and intercepts) of A-MUD and USVSEG. As R2 notes, the difference between slopes (and intercepts) is not reliable when computed using two sets of data. A permutation test (see supplementary file 3) showed that there would not be much difference between the estimated duration of USVs by A-MUD and USVSEG. This explanation is added in the lines 495-501 and the supplementary file 3.**

- The abstract states: “We also did a systematic comparison of existing classification algorithms, where we found the need to develop a new method for automating the classification of USVs using supervised classification, bootstrapping on Gammatone Spectrograms, and Convolutional Neural Networks algorithms with Snapshot ensemble learning (BootSnap)”; Far from a systematic comparison of existing classification algorithms,

**We agree and therefore we changed the beginning of the sentence above to “For automating the classification of USVs, we developed a new method, called BootSnap, using supervised classification, bootstrapping on Gammatone Spectrograms, and Convolutional Neural Networks algorithms with Snapshot ensemble learning. It successfully classified calls into 12 types, including a new class of false positives that is useful for detection refinement.” Please note that at the time of writing, DSQ was the only method to our knowledge that provided public access to the classifier weights needed to perform the evaluation. The other method, VocalMat, was only released (published) after we completed our manuscript. We included this method in our old manuscript (lines 859-871) by reporting the performance of VocalMat on its test data. It was not possible to compare other models that did not provide their weights (e.g., (Premoli et al., 2021)). However, we would like to stress that the architecture of these models is very similar to the one used in DSQ and VocalMat.**

- a single CNN architecture is tested with a single preprocessing pipeline with all combinations of four resampling approaches and four Ensembling methods.

**We have evaluated the model for different values of its hyperparameters and architecture to achieve the best performance, but we did not report it in the first manuscript for the sake of brevity. In the revised version, and according to the suggestion of both R1 and R2, we added a comparison of the performance of the baseline model (without output ensemble and input resample) for different hyperparameters and different network architecture, shown in the supplementary table 4.**

- The combination of methods tested -- SMOTEENN, bootstrapping, XGBM, Snapshot ensembles -- are not very well motivated given that they add considerable complexity.

**For clarification, we added the following lines to the text:**

**Lines 321-324: A spectrogram (the squared modulus of the STFT) is often used for the analysis of USVs and for machine learning approaches (Coffey et al., 2019; Premoli et al., 2021). But the problem is that spectrograms lead to high computational demands and, because of redundancy, they pose high risks of model overfitting.**

**Lines 212-221: Neural networks are being increasingly used for mouse USVs classification (Abbasi et al., 2019; Coffey et al., 2019; Premoli et al., 2021). Machine learning is an iterative method and it can fail to find the most effective weights for classification, however, because the algorithm takes a path that reduces the error and this can lead to focusing on specific weights, which may not be very useful.** **Becoming trapped in a local minimum is a common problem, and it can reduce the generalizability of a classifier (Huang et al., 2017). It can be overcome by using ensemble machine learning methods (Zhou, 2012), a procedure that uses multiple algorithms and the final output is obtained from combining the outputs of these models. This approach makes it possible to obtain a model with better performance than any of its component models and it allows for more flexible structures, though developing ensembles requires additional training time.**

**Lines 419 and 420: The use of the Snapshot Ensemble does not add complexity to the classifier, whereas it does help to take advantage of ensemble learning without needing to train additional models.**

**Lines 393 and 394: Here, we used bootstrap to increase (decrease) the randomness (variance) during the model development.**

**They all lead to increasing the generalizability of the model.**

**The prevalent problem with mouse USVs data is that they are imbalanced in different classes, so it is important to investigate the effect of data oversampling through methods such as SMOTEENN on classifier performance (lines 389-391 and 401-404). According to personal experience, one of the most successful methods in ensembling the output of several models is the XGB method. Therefore, in this study, it was implemented in the developed model (lines 434-441). However, according to our results, neither of these methods did improve the performance of the classifier.**

**lines 548 and 549: Using the XGB output ensembling for bootstrapped data and SMOTEENN increased model complexity and did not improve the performance of the classifier.**

- and there is no “standard” CNN model that is tested as a baseline. There are perhaps a few standard CNN models that could serve as a baseline, for example a single model trained with class-weighted cross entropy loss on the original training set.

**In the old manuscript, the baseline model was the one with the requested specifications of R2, but with the difference that the Snapshot ensemble was also applied in that model (line 551 and 552 in the old manuscript). In the revised manuscript, the baseline model does not include Snapshot learning anymore. It is now based on a constant learning rate (lines 509-512).**

- It is clear from Figure 6 that the bootstrapping method increases average F1 scores by a modest 2-3%, but the effect of snapshot learning is never determined because it is present in every model.

**As mentioned before, the baseline without using Snapshot learning is now added in the figures 5 and 6. Also, the related description is added in the lines 513-515.**

- Other useful comparisons would include predictions using standard spectrograms or various acoustic features as input instead of Gammatone spectrograms. I did not find the techniques in this section novel or the results particularly notable.

**Based on the literature (Premoli et al., 2021) and our experiments, the use of the feature leads to poor performance of the model, and so we did not use it here (lines 204-206). Regarding the use of spectrograms as input, we developed this model to satisfy our curiosity. Our results show that the performance of the model fed by spectrograms has a lower performance on the evaluation data compared to the Gammatone-based models (59% vs 65%). To avoid confusing the reader due to the multiplicity of models developed, we do not discuss this model in our revised manuscript.**

- In the comparison to DeepSqueak's supervised classification, it is not clearly stated whether the classification network's parameters are trained on the training set or kept at the default values. If the parameters were not trained, then I do not believe this is a fair comparison to DeepSqueak. If they were trained, I am surprised by its poor performance (for example, the F1 score of 0 on the 'c' syllable).

**Neither this model nor BootSnap have been trained on the evaluation data. As discussed above, this is because the main purpose of this study is to find a tool that has the highest efficiency with the least user intervention. Therefore, this is a fair comparison. In particular, our evaluation data also included USVs from laboratory mice that are used to develop DSQ (see (Chabout et al., 2015)). To our knowledge, there is no similarity between BootSnap model development data and the evaluation data. As shown in Figure 8, the lab mouse data (i.e., EV_lab) is very different from the development data used in BootSnap.**

**Regarding the poor performance of DSQ in class ‘c’, our model also showed one of its poorest performances in this class.**

Minor Comments

--------------

- The final paragraph provides an interesting and useful discussion of the perceptual validity of syllable categories. There are also similar uncertainties that can be raised on a purely signal-level basis that could diminish a researcher's interest in a categorical description of syllables (Coffey et al. 2017, Figure S6; Sainburg, Thielk, & Gentner 2020; Goffinet et al. 2021). Discrete or continuous, one would hope that a syllable's description is related to biology in some way -- for example, predictive of social context, sex, age, genotype, or individual. Results of this sort could be more convincing than predictions of experimenter-defined labels that are known to be only loosely biologically relevant.

**Thanks for the suggestion. The discussion on signal-level basis** **classification of** **USVs is added in lines 63-75.**

- line 211: “Develop the first classifier based on the CNNs algorithm”; Gradient descent is the algorithm used to train a CNN.

**We thank the reviewer for this comment. Considering this comment and the one on the next page, we changed line 228 and 229 to “Develop a CNN Snapshot Ensemble classifier based on the stochastic gradient descent algorithm”.**

- Figure 9: what is being represented by t-SNE? Vectorized spectrograms? Gammatone filtered spectrograms?

**They are the vectorized Gammatone spectrograms. This point has now been added in the line 670: “….** **on vectorized GSs from both the DEV and EV datasets shown in Figure 8**….”

- Sections 2.2.1 and 2.2.1 are all standard techniques and could be put in a supplement.

**Done.**

- Line 443: "These P-values assess whether the coefficients are significantly different than zero". Should be different than 0 for b0 and different from 1 for b1.

**This is correct, and we thank the reviewer for this comment. As requested by R1, the p-value computed based on t-distribution was replaced with the p-value based on a permutation test. Please check the lines 495-498 and supplementary file 2 (section “statistical tests”).**

- Line 664: "These results again show how the performance of BootSnap depends upon the type of USV, and that pooling certain classes results in better accuracy." Is this effect driven primarily by the number of classes? For example, if Gaussian noise were clustered by k-means with 12 classes and then the labels of different classes were combined to form smaller numbers of classes, I would expect the F1 scores of classifiers to increase.

**This is correct. In the revised manuscript (lines 597-603 and 631-633), we stated that the purpose of this work is only to provide a basis for comparing our work with other studies that have selected a different number of classes from what we have chosen (12 classes). In addition, this section provides useful information about which classes are the best candidate for pooling.**

- Figure 3 typo: "Modul" -> "Module"

**Done.**

- Line 209: "... we aimed at the following principles: (1) Develop the first classifier based on the CNNs algorithm..." Both Ivanenko et al. (2020, Plos CB) and Steinfath et al. (2021, bioRxiv) present CNN classifiers for mouse vocalizations. For birdsong, there is also TweetyNet (Cohen et al. 2020, bioRxiv), which may be adaptable to USVs.

**In the first version, the emphasis of this sentence was on the classification of low-SNR data. We meant that our model is the first CNN-based classifier to be trained on low-SNR data, and has better performance for such data. In the previous studies only high-SNR data were used. To remove any ambiguity, we reworded the above sentence as follows: “Develop a CNN Snapshot Ensemble classifier based on the stochastic gradient descent algorithm, which is accurate even with noisy (low-SNR) data”.**

Reviewer #3:

- The authors systematically compare the performance of four methods to detect rodent ultrasonic vocalizations (USV), while also providing evidence of the success of their own USV automation method, BootSnap. The manuscript is well written, providing a thorough review of the literature in a clear manner. The abstract and introduction clearly state the relevance and importance of the research aims, while also providing details that are helpful for a reader who may implement these methods in the future (e.g. processing time in hours). The author’s third aim (“How well do USV detection tools generalize and perform when using data that differs from the training set (by generalization or out-of-sample error)?”) sets this study apart from the other studies that have compared the performance of USV detection methods in the past (e.g. Coffey et al. 2019), while also expanding on past studies by evaluating the 4 published methods (MUPET, DSQ, A-MUD, and USVSEG) in one robust study (aim #1). I applaud the authors for focusing the manuscript so that the practical applications are clear, particularly addressing the major issues that future researchers will likely face when applying new recordings to these detection methods.
- However, the introduction is lengthy and could be shortened (especially after stating the initial three aims) by moving text to either the methods or discussion sections, depending on the word limit.

**We reduced the length of the introduction by removing literature on classification of USVs. Please check the removed parts in the lines 165-186 and Table 1 (in the old manuscript) in the introduction section These lines have been moved to the Supplementary file 1 and Supplementary table 1.**

- The methodology does target the main questions appropriately, although I cannot comment on the machine learning methods (2.1.4, 2.2.1-2.2.3), as this is not my expertise. The results are presented clearly and logically and appear to be justified by the data. The figures and tables are all appropriate to answer the main aims and the legends are thorough. However, as with the other sections, the results are lengthy and the manuscript could potentially benefit from moving sections to the supplementary material.

**We are grateful for the comments. We moved the Figure 5 and Table 3 and their explanations to the Supplementary file 3. We could not find any way to reduce the length of the classification results without removing critical points. Nevertheless, we tried to reduce the overall length of the manuscript by reducing Method section (sections 2.2.1, 2.2.2, 2.3, and 2.5 of the old manuscript) and moving text to the Supplementary file 2.**

- Ultimately, it is clear that BootSnap outperforms DSQ but it is still not entirely clear how transferable it will be to new data sets, particularly in a wild setting, which is difficult to predict.

**This is an interesting point, though there are no published data on house mouse USVs from the wild or natural conditions. Certainly, this is a topic for future studies. The EV data used for the DSQ and BootSnap comparison are new and are not used during the development of BootSnap. To further emphasize this important point, the following phrase (“even without any re-training with the new data set”) has been added in the lines 29 and 30 to the abstract.**

- The conclusions spelled out in the discussion do respond to each of the main aims and provide even more context by reviewing relevant literature. My only complaint is that the importance of and context for studying USVs is not spelled out, which would improve the manuscript.

**Thanks for the suggestion. We agree and we address this issue in the introduction in the lines 75-80, as it is too important to wait for the discussion.**

- Overall, the manuscript is long but ambitious, providing many important details to answer each of their aims.

**Thank you. We have tried to shorten the manuscript.**

Abbasi, R., Balazs, P., Noll, A., Nicolakis, D., Marconi, M. A., Zala, S. M., & Penn, D. J. (2019). *Applying convolutional neural networks to the analysis of mouse ultrasonic vocalizations*, DOI:<https://doi.org/10.18154/RWTH-CONV-239263>.

Chabout, J., Sarkar, A., Dunson, D. B., & Jarvis, E. D. (2015). Male mice song syntax depends on social contexts and influences female preferences. *Frontiers in behavioral neuroscience, 9*, 76, DOI:<https://doi.org/10.3389/fnbeh.2015.00076>.

Coffey, K. R., Marx, R. G., & Neumaier, J. F. (2019). DeepSqueak: a deep learning-based system for detection and analysis of ultrasonic vocalizations. *Neuropsychopharmacology, 44*(5), 859-868, DOI:<https://doi.org/10.1038/s41386-018-0303-6>.

Concha‐Miranda, M., Hartmann, K., Reinhold, A., Brecht, M., & Sanguinetti-Scheck, J. I. (2020). Play, but not observing play, engages rat medial prefrontal cortex. *European Journal of Neuroscience, 52*(9), 4127-4138.

Fonseca, A. H., Santana, G. M., Bampi, S., & Dietrich, M. O. (2020). Analysis of Ultrasonic Vocalizations from Mice Using Computer Vision and Machine Learning. *bioRxiv*.

Fonseca, A. H., Santana, G. M., Ortiz, G. M. B., Bampi, S., & Dietrich, M. O. (2021). Analysis of ultrasonic vocalizations from mice using computer vision and machine learning. *Elife, 10*, e59161, DOI:<https://doi.org/10.7554/eLife.59161>.

Hernandez-Lallement, J., Attah, A. T., Soyman, E., Pinhal, C. M., Gazzola, V., & Keysers, C. (2020). Harm to others acts as a negative reinforcer in rats. *Current Biology, 30*(6), 949-961. e947.

Huang, G., Li, Y., Pleiss, G., Liu, Z., Hopcroft, J. E., & Weinberger, K. Q. (2017). Snapshot ensembles: Train 1, get m for free. *arXiv preprint arXiv:.00109*.

Marconi, M. A., Nicolakis, D., Abbasi, R., Penn, D. J., & Zala, S. M. (2020). Ultrasonic courtship vocalizations of male house mice contain distinct individual signatures. *Animal Behaviour*, DOI:<https://doi.org/10.1016/j.anbehav.2020.09.006>.

Morales-Navas, M., Castaño-Castaño, S., Pérez-Fernández, C., Sánchez-Gil, A., Teresa Colomina, M., Leinekugel, X., & Sánchez-Santed, F. (2020). Similarities between the effects of prenatal chlorpyrifos and valproic acid on ultrasonic vocalization in infant wistar rats. *International Journal of Environmental Research and Public Health, 17*(17), 6376.

Odent, P., Creemers, J. W., Bosmans, G., & D’Hooge, R. (2021). Spectrum of social alterations in the Neurobeachin haploinsufficiency mouse model of autism. *Brain Research Bulletin, 167*, 11-21.

Paraouty, N., Rizzuto, C. R., & Sanes, D. H. (2021). Dopaminergic signaling supports auditory social learning. *Scientific Reports, 11*(1), 1-13.

Premoli, M., Baggi, D., Bianchetti, M., Gnutti, A., Bondaschi, M., Mastinu, A., Migliorati, P., Signoroni, A., Leonardi, R., & Memo, M. (2021). Automatic classification of mice vocalizations using Machine Learning techniques and Convolutional Neural Networks. *PloS one, 16*(1), e0244636.

Sanchez, W. N., Pochapski, J. A., Jessen, L. F., Ellenberger, M., Schwarting, R. K., Robinson, D. L., Andreatini, R., & Da Cunha, C. (2021). Diazepam attenuates the effects of cocaine on locomotion, 50‐kHz ultrasonic vocalizations and phasic dopamine in the nucleus accumbens of rats. *British Journal of Pharmacology*.

Vogel, A. P., Tsanas, A., & Scattoni, M. L. (2019). Quantifying ultrasonic mouse vocalizations using acoustic analysis in a supervised statistical machine learning framework. *Scientific reports, 9*(1), 8100, DOI:<https://doi.org/10.1038/s41598-019-44221-3>.

Wu, X., & Zhu, X. (2008). Mining with noise knowledge: error-aware data mining. *IEEE Transactions on Systems, Man, and Cybernetics-Part A: Systems and Humans, 38*(4), 917-932, DOI:<https://doi.org/10.1109/CIS.2007.7>.

Zala, S. M., Reitschmidt, D., Noll, A., Balazs, P., & Penn, D. J. (2017). Automatic mouse ultrasound detector (A-MUD): A new tool for processing rodent vocalizations. *PloS one, 12*(7), e0181200, DOI:<https://doi.org/10.1371/journal.pone.0181200>.

Zhou, Z.-H. (2012). *Ensemble methods: foundations and algorithms*: CRC press.
