# Supplementary material for: Capturing the songs of mice with an improved detection and classification method for ultrasonic vocalizations (BootSnap)
Source: PLoS Comput Biol. 2022 May 12;18(5):e1010049. doi: 10.1371/journal.pcbi.1010049 (PMC9098080; doi:10.1371/journal.pcbi.1010049)
Supplement: Attachment — Submitted filename: Response to reviewers.docx [file pcbi.1010049.s024.docx]

**Dear Prof. Stowell and dear Prof. Komarova;**

**We would like to thank you and the reviewers for your thoughtful comments about our revised manuscript. Here, we address all of the comments, and highlight changes we made in our revised manuscript (please see below).**

**Sincerely,**

Reviewer #1: I am satisfied with how the authors addressed the issues that I raised, and with the additional analyses.

- In relation to the points made by R2, I would like to see a good-faith effort by the authors to compare trained models for DSQ and BS on the evaluation datasets. The comparison of the difference between non-retrained and retrained models will shed light on the out-of-the-box and ultimate potential of both methods on these particular out-of-sample validation sets.

**Thank you for your suggestion. To address this question, we trained the DSQ classifier using DEV data (i.e., a dataset from wild mice, which was also used to develop BootSnap ), and then evaluated its performance. In summary, we found that the DSQ classifier retrained on DEV improved its performance when tested with EV_wild compared to the pretrained DSQ (i.e., the classifier weights obtained from the original DSQ paper). The F1-score after training on DEV and testing with wild mouse evaluation data (EV_wild) increased from 41% to 66% (1.6-fold increase). This result shows the improved performance of DSQ for classifying wild mice after re-training with wild mice data. However, when we evaluated retrained DSQ with laboratory mice (EV-lab), the F1-score did not improve, and it was actually *reduced* (from 49% to 40%; 1.2-fold decline). This interesting result shows that re-training DSQ with DEV dataset impairs its performance for classifying calls of laboratory mice (used for original training of DSQ). In contrast, BootSnap performed better than DSQ in both types of evaluations (and especially for classifying laboratory mice). The F1-scores of BootSnap (trained on DEV) evaluated with laboratory and wild mice were 64% and 67%, respectively. Thus, our new analyses show that the performance of “a retrained DSQ” is not necessarily improved, and that it can be reduced, depending on the training and test set. A retrained DSQ still does not outperform BootSnap, and moreover, BootSnap provides greater generalizability.**

**We updated our results and provided the explanation of them in our revised ms, i.e. line 30 (abstract section), Table 3, lines 657-661, 678-683, 687-689, 780, 781, 787, and 788 (results section), lines 803, 885-888, and 897-910 (Discussion section), supplementary File 2, and supplementary Dataset 5 and 6 are also updated accordingly.**

- In order to make the most of the test sets, I would recommend to test the retrained models under cross validation.

**It is not completely clear what Reviewer 1 is suggesting here, but we considered three possibilities.**

(i) **If Reviewer 1 is requesting a cross-validation of the DEV data, then we already did this (lines 250-252). DEV data were already divided into 8 folds by cross-validation. In each fold, we had three sets of training, validation, and testing sets. Apart from the DEV data, we had the evaluation dataset (EV) that was not used for training the model.**
(ii) **If Reviewer 1 is suggesting that we should perform cross-validation on the combination of EV and DEV data, then this does not make sense in our view. We do not want our model to “see” the EV data at all. If this dataset is used in the training process, then the EV data is not reliable for examining the generalizability of the model.**
(iii) **If Reviewer 1 is suggesting that we should divide only the EV data into multiple folds, because the final F1-score is the average F1-score on those multiple folds, then, in our thinking, this would not provide a new result. Still, we investigated this approach, and, we divided the EV data into 10 folds, and we found that the resulting F1-scores are 47±2% (pretrained DSQ), 49±2% (retrained DSQ), and 67±3% (BootSnap), respectively. As these results do not add anything new to our findings (the average F1-score obtained from EV-wild and EV-lab data are similar to these results), we did not include them in our revised ms; but of course, we can provide them if the editor prefers.**

Reviewer #2:

- 1) I agree that it would be valuable and convenient to have automated tools that work "out of the box," with no experiment-specific model training. The clarification of this desideratum in the abstract and introduction is helpful.

**We thank the reviewer for this positive comment, and we agree that clarification of this point in the Abstract and Introduction is important.**

- The results in Figure 4 suggest that USVSEG and A-MUD have high enough true positive rates (>96% for three unseen datasets) to be used out of the box for syllable detection for many applications, provided that additional steps are taken to remove false positives.

**We thank the reviewer for this positive comment. Note that we tested these methods for four unseen datasets. The four recordings were compared for each method i.e., laboratory mice vs wild mice for both DEV (i.e., DEV_1 and EV_wild_1) and EV datasets (i.e., EV_lab_1 and EV_lab_2). Please note that none of these data were used to tune A-MUD parameters.**

- However, I do not believe the results for syllable classification are strong enough to merit the use of pre-trained classifiers as the authors propose. If I'm not mistaken, the results in Table 3 indicate how well the pre-trained classifier generalizes to new datasets. The 6-class macro F1 scores here of about 66%, with individual F1 scores as low as 28%, are well below the estimated 90% achieved by manual labels (Table 5).

**We have a very different view of our aims, results, and conclusions. Our ultimate goal is to develop an automated algorithm that is as good or better as a human observer, and we do not claim to have solved this challenging problem fully. We show that none of the existing tools are comparable to humans, including BootSnap, i.e., we acknowledge that the human F1-score in the EV data is higher than the F1-score of the out-of-box model. Nevertheless, we show that our first version of BootSnap outperforms existing models, including DSQ: the performance of BootSnap (67%) was better than DSQ, both pretrained (47%) and retrained (49%). We suggest that the performance of BootSnap can be improved in the future, and we explained how this should be possible. Finally, we emphasized that it is still unclear whether mice can discriminate against all USVs invented by researchers. It is possible that mice use only a few types, and BootSnap works very well for several types of USVs. Based on our results, we have clarified which types of USVs are problematic for automated classification.**

**To prevent any misunderstandings, we included the following paragraph in the Discussion to summarize these points between lines 962-967.**

**“ In summary, our ultimate goal was to develop an automated algorithm that provides an out-of-the-box method whose performance is as good or better than a human observer (manual classification). The human F1-score in EV data was higher than the F1-score of the out-of-the-box Bootsnap model (89.7±1.9% vs 67%, respectively). Although BootSnap does not yet achieve our original aim, this first version provides an advance, as it outperforms other methods, including the state-of-the-art model (DSQ; 47% pretrained and 49% retrained). This leaves room for future research.”**

- 2) Even in the revised manuscript, it is not stated clearly that DeepSqueak is used without fine-tuning the network, in a way that was not evaluated by its developers in the original DeepSqueak paper.

**We apologize, we had the impression that we made this point very clear in the last revision. We now try to state this point even more clearly and we added the following passage to the revised ms (in lines 657-661):**

**“In order to fairly evaluate the performance of the DSQ classifier, we have evaluated both the out-of-the-box (pretrained) and retrained models. In the out-of-the-box model, we have used classifier weights obtained from the original DSQ paper. In the retrained model, we used the classifier weights obtained from training the DSQ classifier using DEV data (see Supplementary File 2).”**

- It is fair to point out, however, that of seven papers using DeepSqueak for syllable detection only two retrained the DeepSqueak detection network, demonstrating that the method is not always used in the best ways in practice. However, of the papers cited that don't fine-tune the network parameters, some perform additional checks were performed to verify the performance (e.g. Hernandez-Lallement et al., 2020).

**We agree that most users apparently have the wrong impression that DSQ provides an out-of-the-box solution and performs as well as manual detection. We hope that we have now clarified this confusion in our revised version to avoid further confusion (lines 501 and 502). Users need to be aware of the limitations of using these tools without re-training and fine-tuning. But this also shows that many users have the wish to be supported by an out-of-the-box solution.**

- For what it's worth, I have used DeepSqueak before and found it fairly straightforward and user-friendly.

**We agree fully. We did not mean to give the impression that this is not the case, and we did not address the user-friendliness of DSQ. We aim to address the out-of-the-box usability of DSQ and its generalizability. To clarify this issue, we added the following text to the discussion section of the ms (lines 874-877):**

**“DSQ is a user-friendly and straightforward tool for analyzing mouse vocalizations, as the user can train it for their data without the need for programming. In this analysis, however, we examined its generalizability and out-of-the-box usability for unseen data, as most users currently use this tool.”**

- Additionally, the authors propose a process in which a network is repeatedly retrained with new user input to cut down on the total amount of user input needed. Even if the authors' intent is to compare out-of-the-box methods, it would be useful as a point of comparison to include a fine-tuned DeepSqueak detection network.

**Based on the reviewer’s suggestion, we have evaluated the DSQ detector performance based on retrained DSQ. We noticed that the DSQ 1.0 (which we have already used for detection and classification) no longer supports the retraining of its detection model. Therefore, and according to the suggestion of the developers of DSQ, we have used the updated version of it (DSQ 3.0.1) for training the DSQ detection model under MATLAB 2021b.**

**We retrained the DSQ detection model based on 2430 USVs from the DEV dataset (excluding DEV_1). We manually corrected the detections, which included the correction of the time and frequency stamp of each USV and the removal of ‘FP’s from the detected segments. We have also tried to keep a reasonable number of segments from all USV types and even included faint and low-SNR USVs. The “duplicate” parameter, which controls the number of duplicates from training images, was set to 2. The network training has been implemented both from scratch and based on the existing trained network (called “Mouse Detector YOLO R2”). Because the detector trained on the pretrained model outperformed the detector trained from scratch, we only reported the results of the first model here. The TPR (FDR) for the data DEV_1, EV_wild_1, EV_lab_1, and EV_lab_2 are 77±2% (1±2%), 87±3% (2±4%), 80% (0%), and 88% (2%), respectively. We kindly ask Reviewer 2 whether we should report these results in the ms.**

**As far as we know, this model has not been evaluated in any peer-reviewed research, and thus we have not included these results in the ms, as it may underestimate this method. Even if we know the highest capability of this detector, the model training to find the best detector is very time-consuming as it is based on trial and error.**

**We stressed that the retrained DSQ detector and classifier is not an out-of-the-box approach. Our tests with the retrained DSQ classifier with wild mice show how its performance substantially improved for EV_wild dataset, as expected. However, the F1-score of the retrained model for EV_lab data was actually reduced, which confirms the need for a generalizable method.**

- 3) Due to the authors' response to my question about training the DeepSqueak classifier, I now realize that the quantitative comparison in Table 3 is not a fair comparison. As stated in the DeepSqueak paper under "Supervised neural network-based classification," the default DeepSqueak classification network was trained using labels produced by an unsupervised k-means clustering algorithm. These clusters were then simply assigned names post-hoc by visually inspecting the corresponding spectrograms. The authors' network, on the other hand, was trained using manual labels produced by the same experts that produced the labels for model evaluation. Therefore, the pre-trained DeepSqueak classifier had no access to manual labels, let alone the labels of the same expert labelers who produced the evaluation labels, unlike the BootSnap classifier. I consider this a major flaw of the manuscript.

**We purposely did not allow the pre-trained DeepSqueak classifier to have access to the manual labels, since our main goal was to check the out-of-the-box supervised generalizability of different methods. However, for the sake of fairness, we also reported the performance of the retrained DSQ classifier.**

**As we explained to Reviewer 1, in our revised version,** **we trained the DSQ classifier using DEV data (i.e., a dataset from wild mice, which was also used to develop BootSnap ), and then evaluated its performance. In summary, we found that the DSQ classifier retrained on DEV improved its performance when tested with EV_wild compared to the pretrained DSQ (i.e., the classifier weights obtained from the original DSQ paper). The F1-score after training on DEV and testing with wild mouse evaluation data (EV_wild) increased from 41% to 66% (1.6-fold increase). This result shows the improved performance of DSQ for classifying wild mice after re-training with wild mice data. However, when we evaluated retrained DSQ with laboratory mice (EV-lab), the F1-score did not improve, and it was actually *reduced* (from 49% to 40%; 1.2-fold decline). This interesting result shows that re-training DSQ with the DEV dataset impairs its performance for classifying calls of laboratory mice (used for original training of DSQ). In contrast, BootSnap performed better than DSQ in both types of evaluations (and especially for classifying laboratory mice). The F1-scores of BootSnap (trained on DEV) evaluated with laboratory and wild mice were 64% and 67%, respectively. Thus, our new analyses show that the performance of “a retrained DSQ” is not necessarily improved and that it can be reduced, depending on the training and test set. A retrained DSQ still does not outperform BootSnap, and moreover, BootSnap provides greater generalizability.**

**We updated our results and provided the explanation of them in our revised ms, i.e. line 30 (abstract section), Table 3, lines 657-661, 678-683, 687-689, 780, 781, 787, and 788 (results section), lines 803, 885-888, and 897-910 (Discussion section), supplementary File 2, and supplementary Dataset 5 and 6 are also updated accordingly.**

**We also updated the results in the section “Comparing BootSnap and DSQ: sensitivity to new classes” (lines 780-788 and 897-910) according to the predictions of the retrained DSQ model. The results show that the retrained DSQ, similar to BootSnap, assigned members of the classes ‘c4’ and ‘c5’ (which are from EV_wild) to jump-included classes (i.e., ‘c2’ and ‘c3’).**

**We want to emphasize that the performance of retrained DSQ is worse than pretrained DSQ for EV-lab. The main reason for this result seems to be the absence of laboratory mice soundfiles in the DEV data. So, this result indicates that DSQ is less generalizable than BootSnap.**

- 4) The addition of the baseline CNN model is an improvement, showing that the combination of snapshot ensembles to the baseline model improves performance. It is unclear whether bootstrapping alone, however, explains the performance of the best classifiers.

**We thank Reviewer 2 for the suggestion and we added the result related to training the model without** **using the snapshot and only based on bootstrap to Figure 5 in the ms. According to this result, bootstrapping increases the F1-score of the baseline model from 68.9±2.3% to 75±1.6%.**

- 5) As the authors note (lines 212-221), neural networks are increasingly being used to classify mouse USVs. I do not believe simply training a new neural network model using low-SNR data (lines 228-229) adds substantial novelty to the methods (DeepSqueak detection networks were trained using artificially low-SNR data). Neither is using Gammatone filters (MUPET uses Gammatone filters). And adding ensemble methods, although possibly useful, is not particularly novel either.

**This is a misunderstanding. We do not claim to have developed a new general machine learning method of general interest to the computer science community. The goal of our research is applied machine learning and to combine existing approaches in a clever and novel way to develop a practical tool for the task at hand (and the topic ‘mouse ultrasonic vocalizations’ has >16,000 citations on Google Scholar).**

**The comment from Reviewer 2 just cited makes it clear that we have combined three different approaches, adapted it to a new type of dataset and so created a novel approach. According to the results, the use of these components has improved the generalizability of the classifier compared to pretrained and retrained DSQ.**

**We changed line 236 and clarified this point as follows: “Apply a CNN Snapshot Ensemble ….“ and added the following sentence (“So, the novelty of BootSnap comes from a clever combination of bootstrapping approach, snapshot ensemble, and Gammatone spectrograms.”) to the discussion (lines 895 and 896).**

- 6) The addition of more hyperparameter choices for the detection algorithms is an improvement and did, indeed, improve detection performance.

**We thank Reviewer 2 for this positive comment.**

- Summary: I think the comparison of USV detection algorithms presented in Figure 3 is a useful demonstration of the abilities of USVSEG and A-MUD compared to MUPET,

**We thank Reviewer 2 for this positive comment.**

- although I have issues with how DeepSqueak is presented as explained above.

**According to this comment, we made some changes and updated some sections (line 30 (abstract section), Table 3, lines 657-661, 678-683, 687-689, 780, 781, 787, and 788 (results section), lines 803, 885-888, and 897-910 (Discussion section), supplementary File 2, and supplementary Dataset 5 and 6) in the ms.**

- The neural network classification method is not particularly novel and the comparison against the pre-trained DeepSqueak network is not a fair one.

**See our comments above. Although we think that we added some novelty to the field with our method, we now revised the ms to make clear that the novelty comes from a clever combination of techniques and not developing a completely new approach (lines 895 and 896).**

- Additionally, the results are not strong enough to justify the "out-of-the-box" classification approach the authors propose.

**For many researchers, an out-of-the-box approach would be the ultimate goal. As Reviewer 2 pointed out, most researchers that use DeepSqueak have the same approach to the problem and we clarified this point in the ms (lines 962-967). This motivates our research as a further step in this direction.**

- The inclusion of inter-observer reliability data was useful in drawing this conclusion and is good practice as well. Overall, despite several improvements of this manuscript over the first submission, I maintain that it is not suitable for publication.

**We hope that the current changes can also change your opinion and we thank Reviewer 2 for the constructive criticism.**
